# Variable-Length Coding with Zero and Non-Zero Privacy Leakage †

**DOI:** 10.3390/e27020124

**Published:** 2025-01-24

**Authors:** Amirreza Zamani, Mikael Skoglund

**Affiliations:** Division of Information Science and Engineering, KTH Royal Institute of Technology, 100-44 Stockholm, Sweden; skoglund@kth.se

**Keywords:** private variable-length coding, minimum entropy coupling, lossless compression, extended functional representation lemma, separation technique

## Abstract

A private compression design problem is studied, where an encoder observes useful data *Y*, wishes to compress them using variable-length code, and communicates them through an unsecured channel. Since *Y* are correlated with the private attribute *X*, the encoder uses a private compression mechanism to design an encoded message C and sends it over the channel. An adversary is assumed to have access to the output of the encoder, i.e., C, and tries to estimate *X*. Furthermore, it is assumed that both encoder and decoder have access to a shared secret key *W*. In this work, the design goal is to encode message C with the minimum possible average length that satisfies certain privacy constraints. We consider two scenarios: 1. zero privacy leakage, i.e., perfect privacy (secrecy); 2. non-zero privacy leakage, i.e., non-perfect privacy constraint. Considering the perfect privacy scenario, we first study two different privacy mechanism design problems and find upper bounds on the entropy of the optimizers by solving a linear program. We use the obtained optimizers to design C. In the two cases, we strengthen the existing bounds: 1. |X|≥|Y|; 2. The realization of (X,Y) follows a specific joint distribution. In particular, considering the second case, we use two-part construction coding to achieve the upper bounds. Furthermore, in a numerical example, we study the obtained bounds and show that they can improve existing results. Finally, we strengthen the obtained bounds using the minimum entropy coupling concept and a greedy entropy-based algorithm. Considering the non-perfect privacy scenario, we find upper and lower bounds on the average length of the encoded message using different privacy metrics and study them in special cases. For achievability, we use two-part construction coding and extended versions of the functional representation lemma. Lastly, in an example, we show that the bounds can be asymptotically tight.

## 1. Introduction

**Figure 1:**
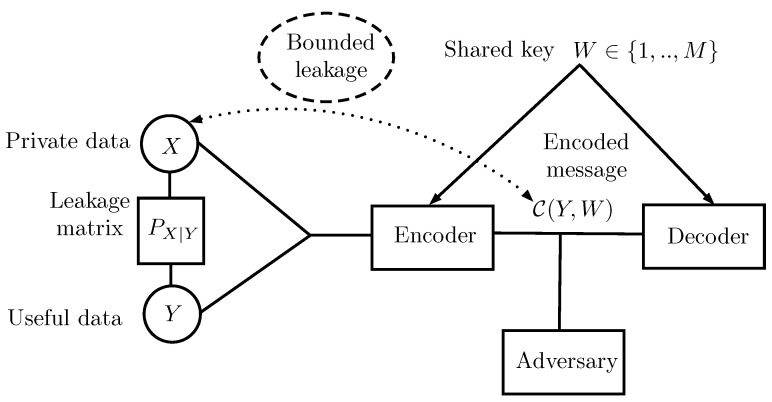
In this work, an encoder wants to compress *Y*, which is correlated with *X* under certain privacy leakage constraints and send it over a channel where an eavesdropper has access to the output of the encoder.

In this work, the random variable (RV) *Y* denotes the useful data and is correlated with the private data denoted by RV *X*. An encoder wishes to compress *Y* and communicates it to a user over an unsecured channel. The encoded message is denoted by the RV C. As shown in Figure 1, it is assumed that an adversary has access to the encoded message C, and wants to extract information about *X*. Moreover, it is assumed that the encoder and decoder have access to a shared secret key denoted by the RV *W* with size *M*. The goal is to design an encoded message C, which compresses *Y*, using a variable-length code with the minimum possible average length that satisfies certain privacy constraints. We utilize techniques used in privacy mechanism and compression design problems and combine them to build such a code C. In this paper, we introduce an approach and apply it to a lossless compression problem.

In this paper, we consider two scenarios, where the privacy leakage is either zero or a bounded leakage is allowed. We refer to these scenarios as perfect and non-perfect privacy scenarios. In the perfect privacy scenario, considering two different cases, we extend previously existing results [1,2]. In the non-perfect privacy scenario, we extend previously existing results [1,2] by generalizing the perfect privacy constraint and allowing non-zero leakage. We use different privacy leakage constraints, e.g., the mutual information between *X* and C equals ϵ, a strong privacy constraint, and bounded per-letter privacy criterion.

### 1.1. Related Works

Recently, the compression and privacy mechanism design problems have received increased attention [1,2,3,4,5,6,7,8,9,10,11,12,13,14,15,16,17,18,19,20,21,22,23,24]. Specifically, in [3], the notion of perfect secrecy was introduced by Shannon, where the public data and private data are statistically independent. Equivocation as a measure of information leakage for information theoretic security was used in [4,5,6]. A rate-distortion approach to information theoretic secrecy was studied in [11]. Maximal leakage was introduced in [12] and used in [13] for the Shannon cipher system. Furthermore, bounds on the privacy–utility trade-off have been derived. The fundamental limits of the privacy–utility trade-off when measuring privacy leakage using estimation-theoretic guarantees were studied in [7]. A privacy mechanism design with total variation as a privacy measure was studied in [8]. A source coding problem with secrecy was studied in [10]. The concept of a privacy funnel was introduced in [14], where the privacy–utility trade-off was studied considering the log-loss as privacy measure and a distortion measure for utility. In both [6,10], privacy–utility trade-offs considering equivocation and expected distortion as measures of privacy and utility were studied.

In [15], the problem of privacy–utility trade-off considering mutual information as measures of both privacy and utility was studied. It was shown that, under perfect privacy, the privacy mechanism design problem can be obtained using a linear program. This was generalized in [25] considering a privacy–utility trade-off with a rate constraint. Moreover, in [15], it was shown that non-zero information about useful data *Y* can be revealed if the kernel (leakage matrix) between private and useful data is not invertible. In [16], the work of [15] was extended by relaxing the perfect privacy assumption, allowing some small bounded privacy leakage. Specifically, privacy mechanisms with a per-letter (point-wise) privacy criterion considering an invertible kernel were designed, allowing a small leakage. This result was extended to a non-invertible leakage matrix in [17].

In [1], an approach to partial secrecy called *secrecy by design* was introduced and applied to two information processing problems: privacy mechanism design and lossless compression. For the privacy design problem, bounds on the privacy–utility trade-off were derived using the functional representation lemma. These results were obtained under a perfect privacy (secrecy) assumption. In [18], the privacy problems considered in [1] were extended by relaxing the perfect secrecy constraint and allowing some leakages. In [20], a privacy–utility trade-off with two different per-letter (point-wise) privacy constraints was studied. The fundamental limits of private data disclosure were studied in [26], where the goal was to minimize the leakage under the utility constraints with non-specific tasks, and it was shown that, under the assumption that the private data are an element-wise deterministic function of useful data, the main problem can be reduced to multiple privacy funnel (PF) problems. Furthermore, the exact solution to each problem was obtained. The concept of lift was studied in [27], which represents the likelihood ratio between the posterior and prior beliefs concerning sensitive features within a dataset. Further privacy leakage measures, such as the differential privacy introduced in [28], (ϵ,δ)-differential privacy [29], local differential privacy [30,31,32,33], maximal leakage [12], and *lift* [27], have been used in the literature.

Moreover, in [1], the problems of fixed-length and variable-length compression were studied, and upper and lower bounds on the average length of an encoded message were derived. These results were obtained under the assumption that the private data were independent of the encoded message, which corresponds to perfect privacy. The minimum entropy coupling concept and a greedy entropy-based algorithm were studied in [34,35,36]. More specifically, in [36], an information spectrum converse was derived and used to find a lower bound on the minimum entropy problem.

### 1.2. Our Contributions

In the perfect privacy scenario, we consider the problem in [1], where for the problem of lossless data compression, strong information theoretic guarantees are provided and fundamental limits are characterized when the private data are a deterministic function of the useful data. In this paper, we improve the bounds obtained in [1].

To this end, we combine the privacy design techniques used in [18], which are based on extended versions of the functional representation lemma (FRL) and strong functional representation lemma (SFRL), as well as the lossless data compression design in [1]. We find lower and upper bounds on the average length of the encoded message C and study them in different scenarios. Considering a specific set of joint distributions for (X,Y), we propose an algorithm with low complexity to find bounds on the optimizer of privacy problems in [18] with zero leakage. We use the obtained results to design C, which achieves tighter bounds compared to [1]. Furthermore, when |X|≥|Y|, we propose a new code that improves on the bounds in [1]. We propose a new design and strengthen the obtained bounds using the minimum entropy coupling concept and a greedy entropy-based algorithm. Finally, in a numerical example, we study the bounds and compare them with [1].

In the non-perfect privacy scenario, our problem is closely related to [1], where we generalize the variable-length lossless compression problem considered in [1] by removing the assumption that *X* and C are independent, i.e., I(X;C(Y,W))=0, and therefore allowing a small leakage.

We find the lower and upper bounds on the average length of the encoded message C and study them in different scenarios. For achievability, we use two-part construction coding. In an example, we show that the obtained bounds can be asymptotically tight. Furthermore, in the case of perfect privacy, the existing bounds found in [1] were improved considering the case where $X=(X_{1},X_{2})$ and $X_{2}$ is a deterministic function of $X_{1}$. The conference versions regarding this paper can be found in [22,23].

Our contribution can be summarized as follows:

(i) In Section 2, we define a private variable-length coding with zero and non-zero leakage problems. We propose a method to deal with the privacy concerns and present privacy–utility trade-offs considering zero and non-zero leakage scenarios.

(ii) In Section 3, we provide a brief overview of the privacy problems in the literature, two essential lemmas, and the minimum entropy coupling concept.

(iii) In Section 4, we find the upper and lower bounds on the trade-offs proposed in Section 2 considering zero and non-zero leakage scenarios. In both scenarios, to find upper bounds, we use the two-part construction coding introduced in [1], which is based on the extended versions of the FRL, the SFRL. We obtain a simple private compression mechanism design that can be optimal in specific scenarios. Moreover, we provide a discussion and comparison of the obtained bounds, with each other and the literature, and the tightness of the bounds is investigated. The results regarding the tightness of the bounds are extended using the concept of the minimum information coupling problem in a zero leakage scenario.

(iv) In Section 5, we present an application in which our method can be applied. Specifically, we consider a cache-aided network and discuss how our results in this paper can be used.

(v) In Section 6, we present an experiment considering the MNIST dataset and we compare our method with previous results in the literature.

Finally, the paper is concluded in Section 7.

## 2. System Model and Problem Formulation

Let $P_{XY}$ denote the joint distribution of discrete random variables *X* and *Y* defined on alphabets X and Y. We assume that the cardinality |X| is finite and |Y| is finite or countably infinite. We represent $P_{XY}$ by a matrix defined on $R^{\left|X|\times |Y\right|}$ and marginal distributions of *X* and *Y* by vectors $P_{X}$ and $P_{Y}$ defined on $R^{\left|X\right|}$ and $R^{\left|Y\right|}$ given by the row and column sums of $P_{XY}$. The relation between *X* and *Y* is given by the leakage matrix $P_{X|Y}$ defined on $R^{\left|X|\times |Y\right|}$. The shared secret key is denoted by the discrete RV *W* defined on {1,…,M} and is assumed to be accessible by both the encoder and decoder. Furthermore, we assume that *W* is uniformly distributed and is independent of *X* and *Y*. A prefix-free code with variable-length and shared secret key of size *M* is a pair of mappings:

$$\begin{array}{c} \left(\mathrm{encoder}\right)\;C:\;X\times Y\times \left\{1,\ldots ,M\right\}\to {\left\{0,1\right\}}^{*}\\ \left(\mathrm{decoder}\right)\;D:\;{\left\{0,1\right\}}^{*}\times \left\{1,\ldots ,M\right\}\to Y. \end{array}$$

The output of the encoder C(Y,W) describes the encoded message. The variable-length code (C,D) is lossless if

(1) $$\begin{array}{c} P(D(C(X,Y,W),W)=Y)=1. \end{array}$$

### 2.1. Non-Zero Privacy Leakage (Non-Perfect Privacy)

Similarly to [1], we define ϵ-private, strongly ϵ-private, and point-wise ϵ-private codes. The code (C,D) is *ϵ-private* if

(2) $$\begin{array}{c} I(C(X,Y,W);X)=\epsilon . \end{array}$$

Moreover, the code (C,D) is *bounded ϵ-private* if

(3) $$\begin{array}{c} I(C(X,Y,W);X)\leq \epsilon . \end{array}$$

Let ξ be the support of C(X,W,Y). For any c∈ξ, let L(c) be the length of the codeword. The code (C,D) is of *(α,M)-variable-length* if

(4) $$\begin{array}{c} E(L(C(X,Y,w)))\leq \alpha ,\;\forall w\in \{1,\ldots ,M\}. \end{array}$$

Finally, let us define the sets $H^{\epsilon }\left(\alpha ,M\right)$ and $H_{b}^{\epsilon }\left(\alpha ,M\right)$, as follows: $H^{\epsilon }\left(\alpha ,M\right)≜\left\{\left(C,D\right):\left(C,D\right)\;\mathrm{is}\;\epsilon \text{-private}\;\mathrm{and}\;\mathrm{of}\;\left(\alpha ,M\right)\text{-variable-length}\right\}$, and $H_{b}^{\epsilon }\left(\alpha ,M\right)≜\left\{\left(C^{\prime },D^{\prime }\right):\left(C^{\prime },D^{\prime }\right)\;\mathrm{is}\;\mathrm{bounded}\;\epsilon \text{-private}\;\mathrm{and}\;\mathrm{of}\;\left(\alpha ,M\right)\text{-variable-length}\right\}$. The private compression design problems can be then stated as follows:

(5) $$\begin{array}{cc} L(P_{XY},M,\epsilon ) & =\underset{\begin{array}{c} \begin{array}{c} \left(C,D\right):\left(C,D\right)\in H^{\epsilon }\left(\alpha ,M\right) \end{array} \end{array}}{\inf}\alpha , \end{array}$$

(6) $$\begin{array}{cc} L^{b}\left(P_{XY},M,\epsilon \right) & =\underset{\begin{array}{c} \begin{array}{c} \left(C,D\right):\left(C,D\right)\in H_{b}^{\epsilon }\left(\alpha ,M\right) \end{array} \end{array}}{\inf}\alpha . \end{array}$$

Clearly, we have $L^{b}\left(P_{XY},M,\epsilon \right)\leq L\left(P_{XY},M,\epsilon \right)$ and $L^{b}\left(P_{XY},M,\epsilon \right)\leq L\left(P_{XY},M,0\right)$.

**Remark** **1.** *Considering ϵ=0, both *(5)* and *(6)* lead to the privacy–compression rate trade-off studied in [1]. In this paper, we generalize this trade-off by considering a non-zero ϵ.*

### 2.2. Zero Privacy Leakage (Perfect Privacy)

We define a perfectly-private code. The code (C,D) is *perfectly-private* if

(7) $$\begin{array}{c} I(C(X,Y,W);X)=0. \end{array}$$

Let ξ be the support of C(X,W,Y) and for any c∈ξ let L(c) be the length of the codeword. The code (C,D) is *(α,M)-variable-length* if (4) holds. Finally, let us define the sets H(α,M) as follows:

H(α,M)≜{(C,D):(C,D)isperfectly-privateand(α,M)-variable-length}. The private compression design problems can be stated as follows:

(8) $$\begin{array}{cc} L^{0}\left(P_{XY},M\right) & =\underset{\begin{array}{c} \begin{array}{c} \left(C,D):(C,D)\in H(\alpha ,M\right) \end{array} \end{array}}{\inf}\alpha . \end{array}$$

Furthermore, let us recall the privacy mechanism design problems considered in [19] with zero leakage as follows:

(9) $$\begin{array}{cc} g_{0}\left(P_{XY}\right) & ≜\max_{\begin{array}{c} \begin{array}{c} P_{U|Y}:X-Y-U\\ \;I(U;X)=0, \end{array} \end{array}}I\left(Y;U\right), \end{array}$$

(10) $$\begin{array}{cc} h_{0}\left(P_{XY}\right) & ≜\max_{\begin{array}{c} \begin{array}{c} P_{U|Y,X}:I\left(U;X\right)=0, \end{array} \end{array}}I\left(Y;U\right). \end{array}$$

Finally, we define a set of joint distributions ${\hat{P}}_{XY}$ as follows:

(11) $$\begin{array}{c} {\hat{P}}_{XY}≜\left\{P_{XY}:g_{0}\left(P_{XY}\right)=h_{0}\left(P_{XY}\right)\right\}. \end{array}$$

**Remark** **2.** *The problem *(8)* was studied in [1]. Here, we improve the bounds in [1] in two different cases and obtain more bounds using the concept of the minimum information coupling problem.*

## 3. Preliminaries and Overview of the Privacy Problems

In this section, we provide a brief overview of a few related privacy problems. We then provide essential lemmas and definitions corresponding to the extended functional representation lemma, the extended strong functional representation lemma, and the minimum information coupling problem. Mutual information has been widely used as a privacy leakage measure [1,2,14,15,18,21,26]. Specifically, in [21], the following problem was addressed

(12) $$\begin{array}{cc} g_{\epsilon }\left(P_{XY}\right) & ≜\underset{\begin{array}{c} \begin{array}{c} P_{U|Y}:X-Y-U\\ \;I(U;X)\leq \epsilon , \end{array} \end{array}}{\sup}I\left(Y;U\right), \end{array}$$

where in ([21], Lemma 1), the lower and upper bounds on $g_{\epsilon }\left(P_{XY}\right)$ were derived. Considering perfect privacy, i.e., ϵ=0, $g_{0}\left(P_{XY}\right)$ can be obtained by solving a linear program ([15], Theorem 1). This implies that the optimal mapping is the solution to a linear program. Furthermore, it has been shown that, to find the optimal privacy-preserving mapping, it is sufficient to consider *U* such that $|U|\leq \mathrm{null}(P_{X|Y})+1$. However, solving the linear program suggested by [15] can become challenging when the size of Y or X increases. Necessary and sufficient conditions for achieving non-zero utility when the private data are hidden, i.e., $g_{0}\left(P_{XY}\right)>0$, were derived in [37,38]. It was shown that $g_{0}\left(P_{XY}\right)>0$ if and only if the rows of $P_{X|Y}$ are linearly dependent. This result was demonstrated and generalized in [15], e.g., see ([15], Proposition 1). Moreover, considering an observable private data scenario, necessary and sufficient conditions for attaining non-zero utility, i.e., $h_{0}\left(P_{XY}\right)>0$, were derived in ([1], Theorem 5), where

(13) $$\begin{array}{cc} h_{\epsilon }\left(P_{XY}\right) & ≜\underset{\begin{array}{c} \begin{array}{c} P_{U|Y,X}:I\left(U;X\right)\leq \epsilon , \end{array} \end{array}}{\sup}I\left(Y;U\right). \end{array}$$

It has been shown that $h_{0}\left(P_{XY}\right)>0$ if and only if *Y* is not a deterministic function of *X*, i.e., H(Y|X)>0.

Next, we present a brief overview of the linear program proposed by [15] that attains $g_{0}\left(P_{XY}\right)$. To do so, let β be a vector in $R^{\left|Y\right|}$. It is shown that $\beta \in \mathrm{Null}(P_{X|Y})$ if and only if β∈Null(M), where $M\in R^{\left|X|\times |Y\right|}$ is constructed as follows: Let *V* be the matrix of right eigenvectors of $P_{X|Y}$, i.e., $P_{X|Y}=U\Sigma V^{T}$ and $V=[v_{1},\;v_{2},\;\ldots ,\;v_{\left|Y\right|}]$, then *M* is defined as

$$\begin{array}{c} M≜{\left[v_{1},\;v_{2},\ldots ,\;v_{\mathrm{rank}(P_{X|Y})}\right]}^{T}. \end{array}$$

Furthermore, if $\beta \in \mathrm{Null}(P_{X|Y})$, then $1^{T}\beta =0$. As shown in [15], for every u∈U, if the Markov chain X−Y−U holds and I(X;U)=0, then the vector $P_{Y|U=u}$ belongs to the following convex polytope S

$$\begin{array}{c} S=\left\{y\in R^{\left|Y\right|}|My=MP_{Y},\;y\geq 0\right\}. \end{array}$$

Using this, we have

(14) $$\begin{array}{c} X-Y-U,\;I\left(X;U\right)=0↔P_{Y|U=u}\in S,\;\forall u, \end{array}$$

which results in the following equivalency

(15) $$\begin{array}{c} \min_{\begin{array}{c} \begin{array}{c} P_{U|Y}:X-Y-U\\ I(X;U)=0 \end{array} \end{array}}H\left(Y|U\right)=\min_{\begin{array}{c} \begin{array}{c} P_{U},\;P_{Y|U=u}\in S,\;\forall u\in U,\\ \sum_{u}P_{U}\left(u\right)P_{Y|U=u}=P_{Y}, \end{array} \end{array}}H\left(Y|U\right), \end{array}$$

where $P_{U}$ defined on $R^{\left|U\right|}$ is the marginal distribution of *U*. Finally, let $P_{Y|U=u}^{*},\;\forall u\in U$ be the minimizer of H(Y|U) over the set $\left\{P_{Y|U=u}\in S,\;\forall u\in U|\sum_{u}P_{U}\left(u\right)P_{Y|U=u}=P_{Y}\right\}$, then, $P_{Y|U=u}^{*}\in S$ for all u∈U must belong to the extreme points of $S_{u}$. Furthermore, it is sufficient to consider $\mathrm{null}(P_{X|Y})+1$ extreme points of S. Using the previous result, the solution to the minimization in (15) can be obtained in two phases: in phase one, the extreme points of set S are identified, while in phase two, proper weights over these extreme points are obtained to minimize the objective function. As argued in [15], the extreme points of S are the basic feasible solutions of S. Basic feasible solutions of S can be found in the following manner. Let Ω be the set of indices which correspond to $\mathrm{rank}(P_{X|Y})$ linearly independent columns of *M*, i.e., $\left|\Omega |=\mathrm{rank}(P_{X|Y}\right)$ and Ω⊂{1,…,|Y|}. Let $M_{\Omega }\in R^{\mathrm{rank}\left(P_{X|Y}\right)\times \mathrm{rank}\left(P_{X|Y}\right)}$ be the sub-matrix of *M* with columns indexed by the set Ω. Then, if all elements of the vector $M_{\Omega }^{-1}MP_{Y}$ are non-negative, the vector $V_{\Omega }^{*}\in R^{\left|Y\right|}$, which is defined in the following, is a basic feasible solution of S. Assume that $\Omega =\{\omega_{1},\ldots ,\omega_{\mathrm{rank}(P_{X|Y})}\}$, where $\omega_{i}\in \left\{1,\ldots ,|Y|\right\}$ and all elements are arranged in an increasing order. The $\omega_{i}$-th element of $V_{\Omega }^{*}$ is defined as the *i*-th element of $M_{\Omega }^{-1}MP_{Y}$, i.e., for $1\leq i\leq \mathrm{rank}(P_{X|Y})$, we have

(16) $$\begin{array}{c} V_{\Omega }^{*}\left(\omega_{i}\right)=\left(M_{\Omega }^{-1}MP_{Y}\right)\left(i\right). \end{array}$$

The other elements of $V_{\Omega }^{*}$ are set to zero. Thus, by using the same arguments as in [15], we obtain the following corollary.

**Corollary** **1.** *Let $S^{*}$ be a set that contains the extreme points of S. If all elements of the vector $M_{\Omega }^{-1}MP_{Y}$ are non-negative, then $V_{\Omega }^{*}\in S^{*}\subseteq S$.*

In the next proposition, we show two properties of each vector inside $S^{*}$.

**Proposition** **1.** *Let $\Omega \subset \left\{1,\ldots ,|Y|\},\;|\Omega \right|=\mathrm{rank}\left(P_{X|Y}\right)$. For every Ω, we have $1^{T}\left(M_{\Omega }^{-1}MP_{Y}\right)=1$.*

**Proof.**  Consider the set $S^{1}=\left\{y\in R^{\left|Y\right|}|My=MP_{Y}\right\}$. Any element in $S^{1}$ has sum elements equal to one, since we have $M\left(y-P_{Y}\right)=0\Rightarrow y-P_{Y}\in \mathrm{Null}\left(P_{X|Y}\right)$, we obtain $1^{T}\left(y-P_{Y}\right)=0\Rightarrow 1^{T}y=1$. The basic solutions of $S^{1}$ are $W_{\Omega }^{*}$ defined as follows: Let $\Omega =\{\omega_{1},\ldots ,\omega_{\mathrm{rank}(P_{X|Y})}\}$, where $\omega_{i}\in \left\{1,\ldots ,|Y|\right\}$, then, $W_{\Omega }^{*}\left(\omega_{i}\right)=M_{\Omega }^{-1}MP_{Y}\left(i\right),$ and other elements of $W_{\Omega }^{*}$ are zero. Thus, the sum over all elements of $M_{\Omega }^{-1}MP_{Y}$ equals one, since each element in $S^{1}$ has to sum to one.  □

After finding all extreme points of the set S, for the second phase, we proceed as follows. Assume that all extreme points of the set S are denoted by $\alpha_{1},\alpha_{2},\ldots ,\alpha_{K}$, which were obtained in the first phase. Then, using the equivalency in Theorem (15), we have

(17) $$\begin{array}{cc} \; & g_{0}\left(P_{XY}\right)=H\left(Y\right)-\min_{\begin{array}{c} \begin{array}{c} \beta :\\ \beta \in R^{K},\;\beta \geq 0\\ {}[\alpha_{1}\;\alpha_{2}\;\ldots \alpha_{K}]\beta =P_{Y} \end{array} \end{array}}\left[H\left(\alpha_{1}\right)\;H\left(\alpha_{2}\right)\;\ldots H\left(\alpha_{K}\right)\right]\beta , \end{array}$$

where $H(\alpha_{i})$ is the entropy of the distribution vector $\alpha_{i}$.

In this work, considering the zero leakage scenario, to design the code, we use the properties of the privacy problems defined in (12) and (13).

Next, we recall two important lemmas from [18].

**Lemma** **1.** *(Extended Functional Representation Lemma (EFRL)): For a pair of RVs (X,Y) distributed according to $P_{XY}$ supported on alphabets X and Y, respectively, where |X| is finite and |Y| is finite or countably infinite, and any 0≤ϵ<I(X;Y), there exists a RV U defined on U such that the leakage between X and U is equal to ϵ, i.e., we have*

$$\begin{array}{c} I(U;X)=\epsilon , \end{array}$$

*Y is a deterministic function of (U,X), i.e., we have*

$$\begin{array}{c} H(Y|U,X)=0, \end{array}$$

*and $\left|U\right|\leq \left[|X|(|Y|-1)+1\right]\left[|X|+1\right].$*

**Lemma** **2.** *(Extended Strong Functional Representation Lemma (ESFRL)): For a pair of RVs (X,Y) distributed according to $P_{XY}$ supported on alphabets X and Y, respectively, where |X| is finite and |Y| is finite or countably infinite with I(X,Y)<∞, and any 0≤ϵ<I(X;Y), there exists a RV U defined on U such that the leakage between X and U is equal to ϵ, i.e., we have*

$$\begin{array}{c} I(U;X)=\epsilon , \end{array}$$

*Y is a deterministic function of (U,X), i.e., we have*

$$\begin{array}{c} H(Y|U,X)=0, \end{array}$$

*I(X;U|Y) can be upper bounded as follows:*

$$\begin{array}{c} I\left(X;U|Y\right)\leq \alpha H\left(X|Y\right)+\left(1-\alpha \right)\left[\log(I(X;Y)+1)+4\right], \end{array}$$

*and $\left|U\right|\leq \left[|X|(|Y|-1)+2\right]\left[|X|+1\right],$ where $\alpha =\frac{\epsilon }{H(X)}$.*

The main idea in constructing *U*, which satisfies the conditions in Lemmas 1 and 2, is to add a randomized response to the output of the FRL and SFRL, respectively. The output refers to a random variable which is produced by the FRL or the SFRL and satisfies the corresponding constraints. In this work, considering the non-zero leakage scenario, to design the code, we utilize the techniques used in Lemmas 1 and 2. The randomization technique was used in finding fair and private representations of a dataset in [39]. To find fair representations, we use randomization techniques similar to those in Lemmas 1 and 2, which lead to lower bounds. Furthermore, this randomization technique has also been applied in the design of differentially private mechanisms [40,41,42,43]. A similar randomization technique was used in [22,23] to address private compression design problems. Next, we recall the important results regarding the upper and lower bounds on the minimum entropy coupling as obtained in [34,35,36]. First, we recall the definition of the minimum entropy coupling problem using ([36], Def. 2).

**Definition** **1.** *(minimum entropy coupling) For a set of PMFs $\left\{P_{1},\ldots ,P_{M}\right\}$, the minimum Shannon entropy coupling is*

(18) $$\begin{array}{c} H^{*}\left(P_{1},\ldots ,P_{M}\right)≜\underset{P_{Y_{1},\ldots ,Y_{M}}:Y_{i}∼P_{i},\forall i}{\inf}H\left(Y_{1},\ldots ,Y_{M}\right), \end{array}$$

*where the infimum is taken over all the joint distributions $P_{Y_{1},\ldots ,Y_{M}}$, where the marginal distribution $P_{Y_{i}}$ equals $P_{i}$ for all i∈{1,…,M}.*

Similarly to [36], for a given joint distribution $P_{XY}$, let the minimum entropy of functional representation of (X,Y) be defined as

(19) $$\begin{array}{c} H^{*}\left(P_{XY}\right)≜\;\;\;\;\;\underset{\begin{array}{c} \begin{array}{c} H(Y|X,U)=0,\;I(X;U)=0 \end{array} \end{array}}{\inf}H\left(U\right). \end{array}$$

**Remark** **3.** *As shown in ([36], Lemma 1), the minimum entropy functional representation and the minimum entropy coupling are related functions. In more detail, $H^{*}\left(P_{XY}\right)$ equals the minimum entropy coupling of the set of PMFs $\left\{P_{Y|X=x_{1}},\ldots ,P_{Y|X=x_{n}}\right\}$, where $X=\{x_{1},\ldots ,x_{n}\}$.*

Let $G_{S}$ be the output of the greedy entropy-based algorithm which was proposed in ([34], Section 3), i.e., $H^{*}\left(P_{XY}\right)\leq H\left(G_{S}\right)$. More specifically, the corresponding algorithm aims to solve (19) but does not achieve the optimal solution in general. Next, we recall a result obtained in [35], which showed that $G_{S}$ is optimal within $\frac{\log e}{e}\approx 0.53$ bits when |X|=2 and is optimal within $\frac{1+\log e}{2}\approx 1.22$ bits when |X|>2. Let $U^{*}$ achieve the optimal solution of (19), i.e., $H\left(U^{*}\right)=H^{*}\left(P_{XY}\right)$.

**Theorem** **1** ([35], Th. 3.4, Th. 4.1, Th. 4.2)**.** *Let $\left(X,Y\right)∼P_{XY}$ and have finite alphabets. When X is binary, we have*

(20) $$\begin{array}{cc} H(Profile) & \leq H\left(U^{*}\right)\leq H\left(G_{S}\right)\leq H\left(Profile\right)+\frac{\log e}{e}\\ & \approx H(Profile)+0.53. \end{array}$$

*Moreover, for |X|>2, we have*

(21) $$\begin{array}{cc} H(Profile) & \leq H\left(U^{*}\right)\leq H\left(G_{S}\right)\\ & \leq H\left(Profile\right)+\frac{1+\log e}{2}\\ & \approx H(Profile)+1.22. \end{array}$$

*Here, Profile corresponds to the profile method proposed in ([35], Section 3).*

Since finding the optimal solution for (19) is challenging, Theorem 1 asserts that $G_{S}$ can be a good approximation for $U^{*}$ that achieves (19). Next, we present the results on lower bounds on $H^{*}\left(P_{XY}\right)$ obtained in a parallel work [36]. The lower bounds were derived using information spectrum and majorization concepts.

**Theorem** **2** ([36], Corollary 2, Th. 2)**.** *Let $\left(X,Y\right)∼P_{XY}$ and have finite alphabets. Let α=1 in ([36], Corollary 2, Th. 2). We have*

(22) $$\begin{array}{c} H\left(\wedge_{x\in X}P_{Y|x}\right)\leq H\left(Q^{*}\right)\leq H\left(U^{*}\right), \end{array}$$

*where ∧ corresponds to the greatest lower bound with respect to majorization and $Q^{*}$ is defined in ([36], Lemma 3).*

**Remark** **4.** *In contrast with [35], the lower bounds in [36] were obtained using Rényi entropy in *(19)*. In this work, we consider Shannon entropy, which is a special case of Rényi entropy.*

**Remark** **5.** *As argued in ([36], Remark 1), for α=1 the (largest) lower bounds obtained in [35,36] match. Thus, using Theorem 1, for binary X, we have*

(23) $$\begin{array}{c} H\left(Q^{*}\right)\leq H\left(U^{*}\right)\leq H\left(G_{S}\right)\leq H\left(Q^{*}\right)+\frac{\log e}{e}, \end{array}$$

*and for |X|>2,*

(24) $$\begin{array}{cc} H(Q^{*}) & \leq H\left(U^{*}\right)\leq H\left(G_{S}\right)\leq H\left(Q^{*}\right)+\frac{1+\log e}{2}. \end{array}$$

*Moreover, in some cases, the lower bound $H(Q^{*})$ is tight, e.g., see ([36], Example 2).*

As discussed in [36], $Q^{*}$ can be obtained by a greedy construction. To do so, let $Q^{*}=\left(q_{1}^{*},q_{2}^{*},\ldots \right)$ with $q_{1}^{*}\geq q_{2}^{*}\geq \ldots$, where $q_{i}=P\left(Q=q_{i}\right)$. Let $P_{Y|X}$ be a matrix with columns $P_{Y|X=x}$, where each is a conditional distribution vector and assume that each column has a descending order (re-order each column). Let $q_{1}^{*}=\min_{x\in X}\left\{\max_{y\in Y}P_{Y|X}\left(y|x\right)\right\}$. In other words, we choose the smallest number in the first row of the matrix $P_{Y|X}$. Next, we subtract $q_{1}^{*}$ from the first row and reorder each column and update the matrix. We then choose the smallest number from the first row of the updated matrix and represent it by $q_{2}^{*}$. We continue this procedure until the summation of $q_{i}^{*}$ reaches one. To see an example, refer to ([36], Example 1).

Finally, we recall the two-part construction coding which was used in [1]. To do so, let us consider the scenario illustrated in Figure 1 where an encoder wishes to compress *Y* and communicates it to a user over an unsecured channel. The encoded message is described by the RV C. As shown in Figure 1, it is assumed that an adversary has access to the encoded message C, and wants to extract information about *X*. Moreover, it is assumed that the encoder and decoder have access to a shared secret key denoted by the RV *W* with size *M*. The goal is to design an encoded message C, which compresses *Y*, using a variable length code with the minimum possible average length that satisfies a zero leakage constraint. The shared secret key is denoted by the discrete RV *W* defined on {1,…,M} and is assumed to be accessible by both the encoder and decoder. Furthermore, we assume that *W* is uniformly distributed and is independent of *X* and *Y*.

**Theorem** **3** ([1], Theorem 8)**.** *Let 0≤ϵ≤H(X) and the pair of RVs (X,Y) be distributed according to $P_{XY}$, and the shared secret key size be |X|, i.e., M=|X|. Then, we have*

(25) $$\begin{array}{c} L^{0}(P_{XY},|X|)\leq \sum_{x\in X}H\left(Y|X=x\right)+1+\left\lceil \log(|X|)\right\rceil , \end{array}$$

*where $\alpha =\frac{\epsilon }{H(X)}$ and if |Y| is finite, we have*

(26) $$\begin{array}{cc} \; & L^{0}(P_{XY},|X|)\leq \left\lceil \log\left(|X|(|Y|-1)+1\right)\right\rceil +\left\lceil \log(|X|)\right\rceil , \end{array}$$

*Finally, if X is a deterministic function of Y, we have*

(27) $$\begin{array}{cc} \; & L^{0}(P_{XY},|X|)\leq \left\lceil \log\left(\left(|Y|-|X|+1\right)\right)\right\rceil +\left\lceil \log(|X|)\right\rceil . \end{array}$$

**Figure 2:**
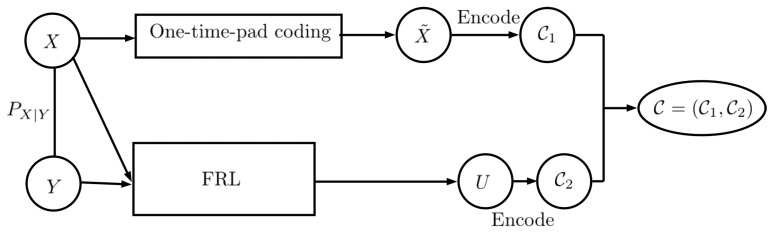
Two-part construction coding: A strategy to send codewords over the channels. We hide the information of *X* using one-time-pad coding, and we then use the FRL to produce *U*.

**Proof.**  Let *W* be the shared secret key with key size M=|X|, which is uniformly distributed over {1,…,M}={1,…,|X|} and independent of (X,Y). As shown in Figure 2, first, the private data *X* are encoded using the shared secret key. Thus, we have

$$\begin{array}{c} \tilde{X}=X+W\;\mathrm{mod}\;\left|X\right|. \end{array}$$

Next, we show that $\tilde{X}$ has a uniform distribution over {1,…,|X|} and $I(X;\tilde{X})=0$. We have

(28) $$\begin{array}{c} H\left(\tilde{X}|X\right)\;=\;H\left(X\;+\;W|X\right)\;=\;H\left(W|X\right)\;=\;H\left(W\right)\;=\;\log\left(|X|\right). \end{array}$$

Furthermore, $H\left(\tilde{X}|X\right)\leq H\left(\tilde{X}\right)$, and combining it with (28), we obtain $H\left(\tilde{X}|X\right)=H\left(\tilde{X}\right)=\log\left(|X|\right)$. For encoding $\tilde{X}$, we use ⌈log(|X|)⌉ bits. Let $C_{1}$ denote the encoded $\tilde{X}$. Next, by using the construction in ([1], Lemma 2), let $U^{*}$ be the output of FRL. Thus, we have

(29)I(U∗;X)=0,(30)H(Y|U∗,X)=0,(31)H(U∗)≤∑xH(Y|X=x)+1.

**Figure 3:**
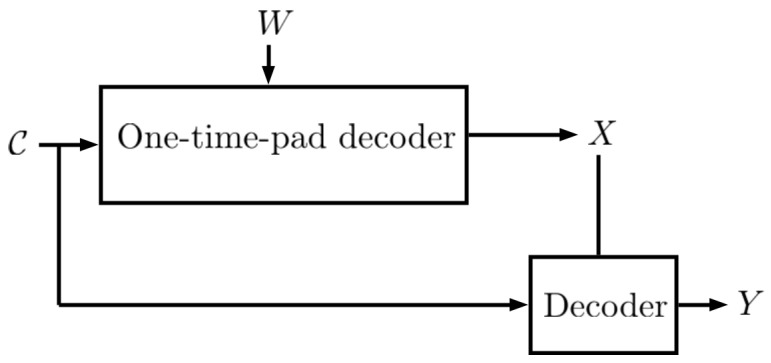
At the receiver side, we first decode *X* using the shared key *W*, then by using the fact that *U* satisfies H(Y|X,U)=0, we can decode *Y* based on *X* and *U*.

We encode $U^{*}$ using any lossless code which uses at most $H(U^{*})+1$ bits and let $C_{2}$ be the encoded message $U^{*}$. We send $C=(C_{1},C_{2})$ over the channel. Thus, (25) can be obtained. Note that, as shown in Figure 3, on the decoder side, we first decode *X* using *W*, by adding |X|−W to $\tilde{X}$, then, *Y* is decoded using the fact that $U^{*}$ satisfies $H(Y|U^{*},X)=0$. Next, we show that if *W* is independent of $\left(X,U^{*}\right)$ we obtain I(C;X)=0. We have

$$\begin{array}{cc} I(C;X) & =I\left(C_{1},C_{2};X\right)=I\left(U,\tilde{X};X\right)\\ & =I\left(U^{*};X\right)+I\left(\tilde{X};X|U\right)=I\left(\tilde{X};X|U^{*}\right)\\ & \overset{\left(a\right)}{=}H\left(\tilde{X}|U^{*}\right)-H\left(\tilde{X}|X,U^{*}\right)\\ & =H\left(\tilde{X}|U^{*}\right)-H\left(X+W|X,U^{*}\right)\\ & \overset{\left(b\right)}{=}H\left(\tilde{X}|U^{*}\right)-H\left(W\right)\\ & \overset{\left(c\right)}{=}H\left(\tilde{X}\right)-H\left(W\right)=\log\left(|X|\right)\;-\;\log\left(|X|\right)\;=\;0 \end{array}$$

where (a) follows from $I(U^{*};X)=0$; (b) follows, since *W* is independent of $\left(X,U^{*}\right)$; and (c) from the independence of $U^{*}$ and $\tilde{X}$. The latter follows, since we have

$$\begin{array}{cc} 0\leq I(\tilde{X};X|U^{*}) & =H\left(\tilde{X}|U^{*}\right)-H\left(W\right)\\ & \overset{\left(i\right)}{=}H\left(\tilde{X}|U^{*}\right)-H\left(\tilde{X}\right)\leq 0. \end{array}$$

Thus, $\tilde{X}$ and $U^{*}$ are independent. Step (i) above follows by the fact that *W* and $\tilde{X}$ are uniformly distributed over {1,…,|X|}, i.e., $H\left(W\right)=H\left(\tilde{X}\right)$. As a summary, if we choose *W* independently of $\left(X,U^{*}\right)$, the leakage to the adversary is zero.  □

## 4. Main Results

Here, we find upper and lower bounds on (5), (6) and (8), and study them in different scenarios.

### 4.1. Zero Leakage Results

In this section, we derive upper and lower bounds on $L^{0}\left(P_{XY},M,\epsilon \right)$ defined in (8). We first study the properties of ${\hat{P}}_{XY}$ and provide a sufficient condition on $P_{XY}$ so that it belongs to the corresponding set. We then find upper bounds on the entropy of the optimizers of $g_{0}\left(P_{XY}\right)$ and $h_{0}\left(P_{XY}\right)$. The obtained bounds help us to find upper bounds on $L^{0}\left(P_{XY},M\right)$. In other words, for $P_{XY}\in {\hat{P}}_{XY}$, we use the solutions of $g_{0}\left(P_{XY}\right)$ and $h_{0}\left(P_{XY}\right)$ to design C; however, in [1], the design of the code was based on the functional representation lemma. Finally, we provide lower bounds on $L^{0}\left(P_{XY},M\right)$, study the bounds in a numerical example, and compare them with [1].

In the next lemma, let C(X,Y) denote the common information between *X* and *Y*, where the common information corresponds to the Wyner [44] or Gács-Körner [45] notions of common information.

**Lemma** **3.** *If C(X,Y)=I(X;Y), then $P_{XY}\in {\hat{P}}_{XY}$.*

**Proof.**  The proof is provided in ([18], Proposition 6) with ϵ=0 and follows similar arguments as the proof of ([46], Theorem 2).  □

**Corollary** **2.** *If X is a deterministic function of Y, then $P_{XY}\in {\hat{P}}_{XY}$, since, in this case, we have C(X,Y)=I(X;Y). Furthermore, this result was also shown in ([18], Proposition 6).*

In the next result, we provide the properties of the optimizers for $g_{0}\left(P_{XY}\right)$ and $h_{0}\left(P_{XY}\right)$.

**Lemma** **4.** *If $g_{0}\left(P_{XY}\right)=h_{0}\left(P_{XY}\right)$, then*

(32) $$\begin{array}{c} g_{0}\left(P_{XY}\right)=h_{0}\left(P_{XY}\right)=H\left(Y|X\right), \end{array}$$

*and both $g_{0}\left(P_{XY}\right)$ and $h_{0}\left(P_{XY}\right)$ have the same set of optimizers. Furthermore, for any optimizer $U^{*}$, we have*

(33)H(Y|U∗,X)=0,(34)I(X;U∗|Y)=0,(35)I(X;U∗)=0.

*Finally, if U satisfies *(33)–(35)*, then it is an optimizer for both $g_{0}\left(P_{XY}\right)$ and $h_{0}\left(P_{XY}\right)$.*

**Proof.**  All the statements can be shown using ([1], Theorem 7) and the key equation as follows:

(36) $$\begin{array}{c} I(U;Y)=I(X;U)+H(Y|X)-I(X;U|Y)-H(Y|X,U). \end{array}$$

 □

**Remark** **6.** *The optimization problems in *(9)* and *(10)* do not have unique optimizers. For instance, let X=f(Y), by using the constructions used in [1,47], we can attain the optimum value.*

Next, we show that if $P_{XY}\in {\hat{P}}_{XY}$, without loss of optimality, we can assume that $|U|\leq \mathrm{null}(P_{X|Y})+1$, where $\mathrm{null}(P_{X|Y})$ corresponds to the dimension of the null space of $P_{X|Y}$.

**Lemma** **5.** *For any $P_{XY}$, we have*

(37) $$\begin{array}{c} g_{0}\left(P_{XY}\right)=\max_{\begin{array}{c} \begin{array}{c} P_{U|Y}:X-Y-U,\\ I(X;U)=0,\\ |U|\leq \;\mathrm{null}(P_{X|Y})+1 \end{array} \end{array}}I\left(Y;U\right). \end{array}$$

*Furthermore, let $P_{XY}\in {\hat{P}}_{XY}$. Then,*

h0(PXY)=g0(PXY)(38)=maxPU|Y:X−Y−UI(X;U)=0,|U|≤null(PX|Y)+1I(Y;U)(39)=maxPU|Y:I(X;U)=0,|U|≤null(PX|Y)+1I(Y;U)

**Proof.**  The proof of (37) is provided in ([15], Theorem 1). It only remains to show the equality (38)=(39). Let $U^{*}$ be an optimizer of (38). Using (37), it is also an optimizer of $g_{0}\left(P_{XY}\right)$ and hence $h_{0}\left(P_{XY}\right)$. Thus, (38)=(39). In other words, for $P_{XY}\in {\hat{P}}_{XY}$, we can assume $|U|\leq \mathrm{null}(P_{X|Y})+1$ in both problems $g_{0}\left(P_{XY}\right)$ and $h_{0}\left(P_{XY}\right)$.  □

Before stating the next result, let us define a set $U^{1}\left(P_{XY}\right)$ and a function $K(P_{XY})$ as follows

(40)U1(PXY)≜{U:Usatisfies(33),(34),(35)}(41)K(PXY)≜minU∈U1(PXY)H(U).

Note that $U^{1}\left(P_{XY}\right)$ can be empty for some joint distributions $P_{XY}$; however, using Lemma 4 when $P_{XY}\in {\hat{P}}_{XY}$ it is the set which contains all the optimizers satisfying $g_{0}\left(P_{XY}\right)=h_{0}\left(P_{XY}\right)$. Furthermore, the function $K(P_{XY})$ finds the minimum entropy of all optimizers satisfying $g_{0}\left(P_{XY}\right)=h_{0}\left(P_{XY}\right)$.

**Lemma** **6.** *Let $P_{XY}\in {\hat{P}}_{XY}$. We have*

(42) $$\begin{array}{c} K\left(P_{XY}\right)\leq \log\left(\mathrm{null}\left(P_{X|Y}\right)+1\right). \end{array}$$

**Proof.**  The proof follows from Lemma 5. Let $U^{*}$ be any optimizer of $g_{0}\left(P_{XY}\right)$ that satisfies $|U^{*}|\leq \mathrm{null}\left(P_{X|Y}\right)+1$. We have $K\left(P_{XY}\right)\leq H\left(U^{*}\right)\leq \log\left(\mathrm{null}\left(P_{X|Y}\right)+1\right)$.  □

**Remark** **7.** *Lemma 6 asserts that when $P_{XY}\in {\hat{P}}_{XY}$, there exist a U that satisfies *(33)–(35)*, and*

(43) $$\begin{array}{c} H\left(U\right)\leq \log(\mathrm{null}\left(P_{X|Y}\right)+1). \end{array}$$

**Remark** **8.** *The upper bound derived in Lemma 6 can be significantly smaller than the upper bound obtained in ([1], Lemma 2). This helps us to find codes that have a lesser average length compared to [1]. Noting that ([1], Lemma 2) asserts that there exists U which satisfies *(33)* and *(35)*, and*

$$\begin{array}{c} |U|\leq |X|(|Y|-1)+1, \end{array}$$

*which results in*

(44) $$\begin{array}{c} H(U)\leq \log(|X|(|Y|-1)+1). \end{array}$$

In the next example, we compare (44) and (43).

**Example** **1.** *Let Y be a deterministic function of X, i.e., Y=f(X), which yields $P_{XY}\in {\hat{P}}_{XY}$ and Y≤|X|. In this case, $\mathrm{null}(P_{X|Y})\leq |X|-1$. We have*

$$\begin{array}{c} \mathrm{null}\left(P_{X|Y}\right)\leq |X|-1<|X|(|Y|-1), \end{array}$$

*which results in*

$$\begin{array}{c} (43)<(44). \end{array}$$

*Furthermore, as |X| and |Y| grow, the gap between (33) and (34) becomes larger. To see another example, let X and Y be represented by $\left(X^{\prime },V\right)$ and $\left(Y^{\prime },V\right)$, where $X^{\prime }$ and $Y^{\prime }$ are conditionally independent given V, then by using [48], we have I(X;Y)=C(X;Y) resulting in $P_{XY}\in {\hat{P}}_{XY}$, where C(X;Y) corresponds to the common information between X and Y. In this case, we have $\mathrm{null}\left(P_{X|Y}\right)\leq \max\left\{|X|,|Y|\right\}-1$. For |X|>2, we have*

$$\begin{array}{c} \mathrm{null}\left(P_{X|Y}\right)\leq \max\{|X|,|Y|\}-1<|X|(|Y|-1), \end{array}$$

*which results in*

$$\begin{array}{c} (43)<(44). \end{array}$$

*Intuitively, when |X| and |Y| are large, *(43)* can be significantly less than *(44)*, since $\mathrm{null}\left(P_{X|Y}\right)\leq \max\left\{|X|,|Y|\right\}-1$ and *(44)* includes multiplication of |X| and |Y|.*

Before stating the next result, we define

(45)AXY≜Py1−Py1|x1…Pyq−Pyq|x1·…·Py1−Py1|xt…Pyq−Pyq|xt∈Rt×q,(46)bXY≜H(Y|x1)−H(Y|X)·H(Y|xt)−H(Y|X)∈Rt,a≜a1·aq∈Rq.

where t=|X| and q=|Y|.

**Theorem** **4.** *Let $P_{XY}\in {\hat{P}}_{XY}$. For any $U\in U^{1}$, we have*

(47)H(Y|X)+minai:AXYa=bXY,a≥0∑i=1qPyiai≤K(PXY)≤(48)H(U)≤H(Y|X)+maxai:AXYa=bXY,a≥0∑i=1qPyiai.

*The upper bound can be strengthened and we have*

(49)K(PXY)≤H(Y|X)+maxai:AXYa=bXY,a≥0,∑i=1qPyiai≤log(null(PX|Y)+1)−H(Y|X)∑i=1qPyiai(50)≤log(null(PX|Y)+1).

*Moreover, when $\mathrm{rank}(A_{XY})=|Y|$ and Y≠f(X), we have $|U^{1}|=1$, and for the unique optimizer we have*

(51) $$\begin{array}{c} K\left(P_{XY}\right)=H\left(Y|X\right)+\sum_{i}P_{y_{i}}a_{i} \end{array}$$

*where $A_{XY}a=b_{XY},a\geq 0$ and $\sum_{i=1}^{q}P_{y_{i}}a_{i}\leq \log\left(\mathrm{null}\left(P_{X|Y}\right)+1\right)-H\left(Y|X\right)$.*

**Proof.**  The proof is provided in Appendix A.  □

**Remark** **9.** *As we outlined earlier, it is shown in [15] that $g_{0}\left(P_{XY}\right)$ can be obtained by a linear program which is presented in Section 3. As we can see in Section 3, each extreme point $\alpha_{i}$ is a vector with size |Y| in which at most $\mathrm{rank}(P_{X|Y})$ elements are non-zero. We recall that β corresponds to the weighting elements in the marginal distribution of U, i.e., $\beta =P_{U}$. The number of extreme points is at most |Y|rank(PX|Y). Hence, if we consider the linear equations $\left[\alpha_{1}\;\alpha_{2}\;\ldots \alpha_{K}\right]\beta =P_{Y}$, the size of the matrix in the system of linear equations, i.e., $\left[\alpha_{1}\;\alpha_{2}\;\ldots \alpha_{K}\right]$, is at most |Y|×|Y|rank(PX|Y) and we have at most |Y|rank(PX|Y) variables in the system. By solving the linear program proposed in [15] we can find the exact value of $K(P_{XY})$ and the conditional distribution $P_{U|YX}$ that attains it. The complexity of the linear program in [15] can grow faster than the exponential functions with respect to |Y|; however, the complexity of our proposed method grows linearly with |Y|. The latter follows since, by considering the system of linear equations in our proposed method $A_{XY}a=b_{XY}$, the size of matrix is |X|×|Y| and we do not need to find all possible extreme points of the set S presented in Section 3. Thus, our proposed upper bound has less complexity compared to the solution in [15]. Furthermore, in a special case where $\mathrm{rank}(A_{XY})=|Y|$, we can find the exact value of $K(P_{XY})$ using our method. One necessary condition for $\mathrm{rank}(A_{XY})=|Y|$ is to have |X|≥|Y|+1, since the summation of rows in $A_{XY}$ equals zero.*

**Remark** **10.** *The optimizer of $g_{0}\left(P_{XY}\right)$ does not help us to build C, since the constraint H(Y|U,X)=0 does not hold in general; however, the optimizer of $h_{0}\left(P_{XY}\right)$ satisfies it. Hence, we consider the cases where the equality $g_{0}\left(P_{XY}\right)=h_{0}\left(P_{XY}\right)$ holds. In this case, H(Y|U,X)=0 and we have tighter bounds on H(U) compared to [1].*

**Remark** **11.** *The upper bound in *(48)* holds for any $U\in U^{1}$; however, the upper bound in *(48)* asserts that there exists $U\in U^{1}$ such that the bound holds. The lower bound in *(47)* can be used to find a lower bound on $L^{0}\left(P_{XY},M\right)$. Similarly to Remark 9, the linear program attaining the lower bound in *(47)* has less complexity than [15].*

**Figure 4:**
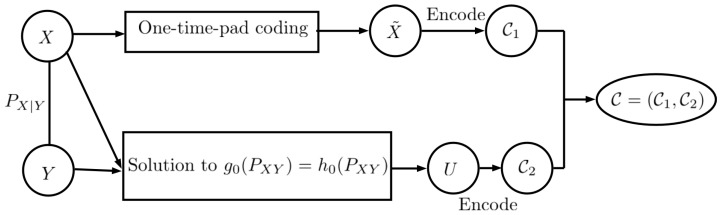
In this work, we use two-part construction coding to send codewords over the channels. The encoder takes *X*, *Y*, and *W* as inputs. In the first step, it hides the information of *X* using one-time-pad coding by adding the secret key *W* to *X* to produce $\tilde{X}$, then, $\tilde{X}$ is encoded to $C_{1}$ using any lossless code. In the second step, the encoder uses the solution of $g_{0}\left(P_{XY}\right)=h_{0}\left(P_{XY}\right)$ to construct *U*, which is independent of *X*. Moreover, *U* satisfies H(Y|U,X)=0, which ensures that *Y* can be recovered from *X* and *U*. Then, *U* is encoded to $C_{2}$ using any lossless code. Finally, $C=(C_{1},C_{2})$ is sent over the channel.

**Figure 5:**
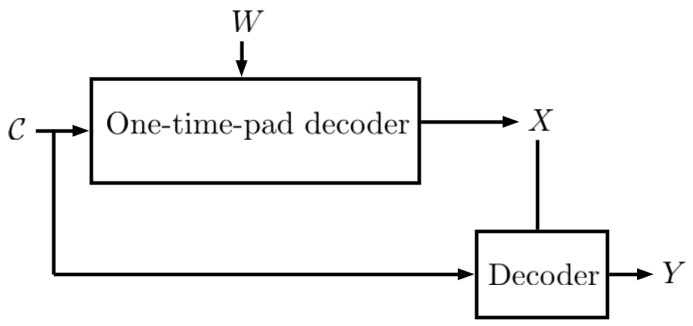
On the receiver side, we first decode *X* using the shared key *W*, then by using H(Y|X,U)=0, we can decode *Y* based on *X* and *U*.

Next, we obtain upper and lower bounds on $L^{0}\left(P_{XY},M\right)$. See Figure 4 and Figure 5.

**Theorem** **5.** *For the pair of RVs (X,Y), let $P_{XY}\in {\hat{P}}_{XY}$ and the shared secret key size be |X|, i.e., M=|X|. Furthermore, let q=|Y| and $\beta =\log(\mathrm{null}\left(P_{X|Y}\right)+1)$. Then, we obtain*

(52)L0(PXY,|X|)≤K(PXY)+1+⌈log(|X|)⌉(53) ≤H(Y|X)+maxai:AXYa=bXY,a≥0,∑i=1qPyiai≤β−H(Y|X)∑i=1qPyiai+1+⌈log(|X|)⌉(54) ≤β+1+⌈log(|X|)⌉

*For any $P_{XY}$, we have*

(55)L0(PXY,|X|)≤minPU|Y,X:I(U;X)=0,H(Y|X,U)=0H(U)+1+⌈log(|X|)⌉(56)≤1+min(∑xH(Y|X=x),log(|X|(|Y|−1)+1)−1)+⌈log(|X|)⌉,

*and if X=f(Y),*

(57) $$\begin{array}{c} L^{0}(P_{XY},|X|)\leq \left\lceil \log(|Y|\;-\;|X|\;+\;1)\;\right\rceil \;+\;\left\lceil \log(|X|)\right\rceil . \end{array}$$

*Finally, for any $P_{XY}$ with |Y|≤|X|, we have*

(58) $$\begin{array}{c} L^{0}(P_{XY},|Y|)\leq \left\lceil \log\left|Y\right|\right\rceil . \end{array}$$

**Proof.**  All upper bounds can be obtained using two-part construction coding [1] and the complete proof is provided in Appendix A.  □

Next, we obtain lower bounds on $L^{0}\left(P_{XY},M\right)$.

**Theorem** **6.** *For any pair of RVs (X,Y) distributed according to $P_{XY}$ and shared key size M≥1, we have*

(59) $$\begin{array}{c} L^{0}\left(P_{XY},M\right)\geq \max_{x}H\left(Y|X=x\right), \end{array}$$

*if X=f(Y),*

(60) $$\begin{array}{c} L^{0}(P_{XY},|X|)\geq \log\left(|X|\right), \end{array}$$

*and the code C does not exist when M<|X| and X=f(Y). Furthermore, considering $P_{XY}\in {\hat{P}}_{XY}$, if we assume the received code C satisfies I(X;C)=0, H(Y|X,C)=0 and X−Y−C, we have*

(61) $$\begin{array}{c} L^{0}\left(P_{XY},M\right)\geq H\left(Y|X\right)+\;\;\;\;\;\min_{a_{i}:A_{XY}a=b_{XY},a\geq 0}\sum_{i=1}^{q}P_{y_{i}}a_{i}. \end{array}$$

**Proof.**  The proofs of (59) and (60) are provided in ([1], Theorem 9) and (61) follows from Theorem 4.  □

**Remark** **12.** *The constraint X−Y−C in the converse bound *(61)* can be motivated by considering the cases where the encoder has no direct access to the private data X. Furthermore, the constraint H(Y|X,C)=0 is stronger compared to the H(Y|X,C,W)=0 that is used to prove *(59)*. Specifically, the constraint needed to prove *(59)* is H(Y|C,W)=0, which results in H(Y|X,C,W)=0.*

### 4.2. Comparison

In this part, we study the bounds obtained in Theorem 5 and compare them with the existing results in [1].

*Case 1:* |Y|≤|X|

Clearly, for any $P_{XY}$ with |Y|≤|X|, the upper bound obtained in (58) improves the bounds in (56) and (55), where (56) was derived in [1]. The latter follows, since in this case we have ⌈log(|Y|)⌉≤⌈log(|X|)⌉.

*Case 2:* $P_{XY}\in {\hat{P}}_{XY}$

In this part, let X=f(Y), which results in $P_{XY}\in {\hat{P}}_{XY}$, see Corollary 1. The upper bound attained by [1] is ⌈log(|Y|−|X|+1)⌉+⌈log(|X|)⌉. Furthermore, we can assume that all elements in the probability vector $P_{X}$ are non-zero, otherwise we can remove them. the In this case, we have

(62) $$\begin{array}{c} \mathrm{null}(P_{X|Y})=|Y|-|X|. \end{array}$$

This follows, since each column in $P_{X|Y}$ contains exactly one non-zero element that equals 1. Moreover, all rows of $P_{X|Y}$ are linearly independent, since each row contains a non-zero element that the other rows do not have. Thus, $\mathrm{rank}(P_{X|Y})=|X|$, which results $\mathrm{null}(P_{X|Y})=|Y|-|X|$. Thus, the upper bound in (54) can be modified to obtain the same bound as (57). Furthermore, the upper bounds (53) and (52) attain less quantities compared to (57), i.e., they can improve the bounds in [1]. Next, in a numerical example, we show that the upper bounds (53) and (52) improve (57).

**Example** **2.** *Let $P_{X|Y}=\left[\begin{array}{cccccc} 1 & 1 & 1 & 0 & 0 & 0\\ 0 & 0 & 0 & 1 & 1 & 1 \end{array}\right]$ and $P_{Y}=\left[\frac{1}{8},\frac{2}{8},\frac{3}{8},\frac{1}{8},\frac{1}{16},\frac{1}{16}\right]$. Clearly, in this case X is a deterministic function of Y. Using the linear program proposed in [15], we obtain a solution as $P_{Y|u_{1}}=\left[0.75,0,0,0.25,0,0\right]$, $P_{Y|u_{2}}=\left[0,0.75,0,0.25,0,0\right]$, $P_{Y|u_{3}}=\left[0,0,0.75,0,0.25,0\right]$, $P_{Y|u_{4}}=\left[0,0,0.75,0,0,0.25\right]$ and $P_{U}=\left[\frac{1}{6},\frac{1}{3},\frac{1}{4},\frac{1}{4}\right]$, which results in H(U)=1.9591 bits. We have $K(P_{XY})+1\leq 2.9591$ and ⌈log(|Y|−|X|+1)⌉=⌈log5⌉=3. Hence, the upper bound *(52)* is strictly lower than *(57)*, i.e., the $U^{*}$ that achieves $g_{0}\left(P_{XY}\right)$ has less entropy than the RV U constructed in ([1], Lemma 1).*

### 4.3. Improving the Bounds Using Greedy Entropy-Based Algorithm

In this part, we use the greedy entropy-based algorithm to generalize the bounds obtained in Theorem 5. In more detail, we use the output of the greedy entropy-based algorithm in Theorem 1 instead of the solution to $g_{0}\left(P_{XY}\right)=h_{0}\left(P_{XY}\right)$. In the next theorem, let $Q^{*}=\left(q_{1}^{*},q_{2}^{*},\ldots \right)$ with $q_{1}^{*}\geq q_{2}^{*}\geq \ldots$, where $q_{i}=P\left(Q=q_{i}\right)$. Let $P_{Y|X}$ be a matrix with columns $P_{Y|X=x}$, and we re-order each column so that each column has a descending order. Let $q_{1}^{*}=\min_{x\in X}\left\{\max_{y\in Y}P_{Y|X}\left(y|x\right)\right\}$, i.e., choose the smallest number in the first row of the matrix $P_{Y|X}$, and then we subtract $q_{1}^{*}$ from the first row and reorder each column and update the matrix. We then choose the smallest number from the first row of the updated matrix and represent it by $q_{2}^{*}$. We continue this procedure until the summation of $q_{i}^{*}$ reaches one.

**Theorem** **7.** *Let the RVs (X,Y) be distributed according to $P_{XY}$ supported on alphabets X and Y, where |X| and |Y| are finite, and let the shared secret key size be |X|, i.e., T=|X|. Let |X|=2, we have*

(63) $$\begin{array}{c} L^{0}\left(P_{XY},2\right)\leq H\left(Q^{*}\right)+\frac{\log e}{e}+2, \end{array}$$

*where $Q^{*}$ is defined in ([36], Lemma 3). When |X|>2, we have*

(64) $$\begin{array}{c} L^{0}(P_{XY},|X|)\leq H\left(Q^{*}\right)+\frac{1+\log e}{2}+1+\left\lceil \log(|X|)\right\rceil , \end{array}$$

**Figure 6:**
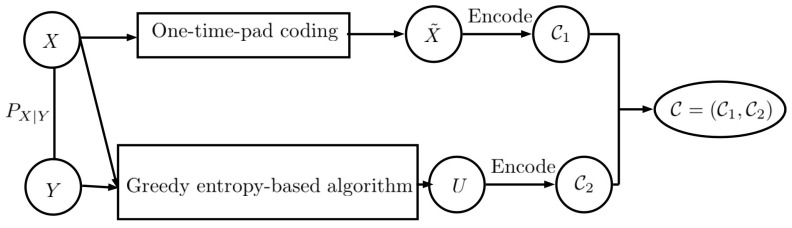
Encoder design: illustration of the achievability scheme of Theorem 7. Two-part code construction is used to produce the response of the server, C. The server sends C over the channel, which is independent of *X*.

**Proof.**  The proof is similar to Theorem 5 and the main difference is to use the minimum entropy output of (19) instead of the solution to $g_{0}\left(P_{XY}\right)=h_{0}\left(P_{XY}\right)$ that is used in two-part construction coding. Similarly to Theorem 5, we use two-part code construction to achieve the upper bounds. As shown in Figure 6, we first encode the private data *X* using one-time pad coding ([2], Lemma 1), which uses ⌈log(|X|)⌉ bits. Next, we produce *U* based on the greedy entropy-based algorithm proposed in [34], which solves the minimum entropy problem in (19). Thus, we have

(65)H(Y|X,U)=0,(66)I(U;X)=0,

and

(67) $$\begin{array}{c} H\left(U\right)\leq H\left(Q^{*}\right)+\frac{\log e}{e}, \end{array}$$

and for |X|>2,

(68) $$\begin{array}{c} H\left(U\right)\leq H\left(Q^{*}\right)+\frac{1+\log e}{2}. \end{array}$$

Thus, we obtain (63) and (64). Moreover, for the leakage constraint, we note that the randomness of one-time-pad coding is independent of *X* and the output of the greedy entropy-based algorithm *U*.

On the user side, we can decode *Y* using (65).  □

**Remark** **13.** *We emphasize that *(63)* and *(64)* can improve *(52)*, since they achieve the minimum in *(56)* within a gap, i.e., achieve $\min_{\begin{array}{c} \begin{array}{c} P_{U|Y,X}:I\left(U;X\right)=0,\\ H(Y|X,U)=0 \end{array} \end{array}}H\left(U\right)$ up to a constant gap that depends on |X|. Furthermore, in contrast with the bounds in Theorem 5, *(63)* and *(64)* can be achieved for any joint distribution $P_{XY}$. However, in the following numerical example, we show that the bounds in Theorem 5 can be tighter than those in Theorem 7.*

**Example** **3.** *Let $P_{X|Y}=\left[\begin{array}{cccccc} 1 & 1 & 1 & 0 & 0 & 0\\ 0 & 0 & 0 & 1 & 1 & 1 \end{array}\right]$ and $P_{Y}=\left[\frac{1}{8},\frac{2}{8},\frac{3}{8},\frac{1}{8},\frac{1}{16},\frac{1}{16}\right]$. Clearly, in this case, X is a deterministic function of Y. Using the linear program proposed in [15], we obtain a solution as $P_{Y|u_{1}}=\left[0.75,0,0,0.25,0,0\right]$, $P_{Y|u_{2}}=\left[0,0.75,0,0.25,0,0\right]$, $P_{Y|u_{3}}=\left[0,0,0.75,0,0.25,0\right]$, $P_{Y|u_{4}}=\left[0,0,0.75,0,0,0.25\right]$ and $P_{U}=\left[\frac{1}{6},\frac{1}{3},\frac{1}{4},\frac{1}{4}\right]$ which results H(U)=1.9591 bits. We have $H\left(U\right)=K\left(P_{XY}\right)\leq 1.9591$. Moreover, we have*

$$\begin{array}{c} P_{Y|X}=\left[\begin{array}{cc} \frac{1}{6} & 0\\ \frac{1}{3} & 0\\ \frac{1}{2} & 0\\ 0 & \frac{1}{2}\\ 0 & \frac{1}{4}\\ 0 & \frac{1}{4} \end{array}\right]. \end{array}$$

*Using the greedy search algorithm, we have $P_{Q*}=\left[\frac{1}{2}\;\frac{1}{4}\;\frac{1}{6}\;\frac{1}{12}\right]$, hence, $H(Q^{*})=1.7296$. Thus,*

$$\begin{array}{c} H\left(Q^{*}\right)+\frac{\log e}{e}=2.2596\geq K\left(P_{XY}\right)=1.9591. \end{array}$$

In the next example, we show that the bounds in Theorem 7 can be tighter than the bounds obtained in Theorem 5.

**Example** **4.** *Let $P_{X|Y}=\left[\begin{array}{cccccc} 1 & 1 & 0 & 0 & 0 & 0\\ 0 & 0 & 1 & 1 & 1 & 1 \end{array}\right]$ and $P_{Y}=\left[\frac{1}{8},\frac{2}{8},\frac{3}{8},\frac{1}{8},0.0925,0.0325\right]$. Similarly to the previous example, X is a deterministic function of Y. Using the linear program proposed in [15], we obtain a solution as $P_{U}=\left[0.185,0.065,0.315,0.25,0.185\right]$, which results in H(U)=2.1696 bits. We have $H\left(U\right)=K\left(P_{XY}\right)=2.182$. Moreover, we have*

$$\begin{array}{c} P_{Y|X}=\left[\begin{array}{cc} \frac{1}{3} & 0\\ \frac{2}{3} & 0\\ 0 & 0.6\\ 0 & 0.2\\ 0 & 0.148\\ 0 & 0.052 \end{array}\right]. \end{array}$$

*Using the greedy search algorithm, we have $P_{Q*}=\left[0.6\;0.2\;0.1333\;0.052\;0.0147\right]$, hence, $H(Q^{*})=1.6053$. Thus,*

$$\begin{array}{c} H\left(Q^{*}\right)+\frac{\log e}{e}=2.136\leq K\left(P_{XY}\right)=2.182. \end{array}$$

### 4.4. Non-Zero Leakage Results

In this section, we derive upper and lower bounds on $L(P_{XY},M,\epsilon )$ and $L^{b}\left(P_{XY},M,\epsilon \right)$, defined in (5) and (6). Next, we study the bounds in different scenarios; moreover, we provide an example that shows that the bounds can be tight. The following lemma helps us to find lower bounds on the entropy of an RV that satisfies I(U;X)=ϵ and H(Y|U,X)=0 considering the shared key.

**Lemma** **7.** *Let the pair (X,Y) be jointly distributed and the RV W be independent of X and Y. Then, if the RV U satisfies I(U;X)=ϵ, and H(Y|U,X,W)=0, then, we have*

(69) $$\begin{array}{c} H\left(U\right)\;\geq \;\max\{L_{1}\left(\epsilon ,P_{XY}\right),L_{2}\left(\epsilon ,P_{XY}\right),L_{3}\left(\epsilon ,P_{XY}\right)\}, \end{array}$$

*where*

(70)L1(ϵ,PXY)=H(Y|X),(71)L2(ϵ,PXY)=minx∈XH(Y|X)+ϵ,(72)L3(ϵ,PXY)=H(Y|X)−H(X|Y)+ϵ,

*and $\min_{x\in X}H\left(Y|X=x\right)$ is the minimum conditional entropy which is non-zero.*

**Proof.**  The proof is provided in Appendix B.  □

In the next two theorems, we provide upper and lower bounds on $L(P_{XY},M,\epsilon )$. The next theorem is a generalization of ([1], Theorem 8) for correlated C and *X*.

**Theorem** **8.** *Let 0≤ϵ≤H(X) and the pair of RVs (X,Y) be distributed according to $P_{XY}$, and the shared secret key size be |X|, i.e., M=|X|. Then, we have*

(73) $$\begin{array}{cc} L(P_{XY},|X|,\epsilon ) & \leq \;\;\;\sum_{x\in X}\;\;H\left(Y|X=x\right)\;+\;\epsilon \;+\;h\left(\alpha \right)\;+\;1+\;\left\lceil \log(|X|)\right\rceil , \end{array}$$

*where $\alpha =\frac{\epsilon }{H(X)}$ and if |Y| is finite, we have*

(74) $$\begin{array}{cc} \; & L(P_{XY},|X|,\epsilon )\leq \left\lceil \log\left(|X|(|Y|-1)+1\right)\left(|X|+1\right)\right\rceil +\left\lceil \log(|X|)\right\rceil , \end{array}$$

*Finally, if X is a deterministic function of Y, we have*

(75) $$\begin{array}{cc} \; & L(P_{XY},|X|,\epsilon )\leq \left\lceil \log\left(\left(|Y|-|X|+1\right)\left[|X|+1\right]\right)\right\rceil +\left\lceil \log(|X|)\right\rceil . \end{array}$$

**Proof.**  The proof is based on ([19], Lemma 5) and the two-part construction coding and is provided in Appendix B.  □

In the next theorem, we provide lower bounds on $L(P_{XY},M,\epsilon )$.

**Theorem** **9.** *Let 0≤ϵ≤H(X) and the pair of RVs (X,Y) be distributed according to $P_{XY}$ supported on alphabets X and Y, where |X| is finite and |Y| is finite or countably infinite. For any shared secret key size M≥1, we have*

(76) $$\begin{array}{cc} \; & L\left(P_{XY},M,\epsilon \right)\geq \max\left\{L_{1}\left(\epsilon ,P_{XY}\right),L_{2}\left(\epsilon ,P_{XY}\right),L_{3}\left(\epsilon ,P_{XY}\right)\right\}, \end{array}$$

*where $L_{1}\left(\epsilon ,P_{XY}\right)$, $L_{2}\left(\epsilon ,P_{XY}\right)$ and $L_{3}\left(\epsilon ,P_{XY}\right)$ are defined in *(70)*, *(71)* and *(72)*. Furthermore, if X is deterministic function of Y, then, we have*

(77) $$\begin{array}{c} L\left(P_{XY},M,\epsilon \right)\geq \log\left(\frac{1}{\max_{x}P_{X}\left(x\right)}\right). \end{array}$$

**Proof.**  The proof is based on Lemma 7 and is provided in Appendix B.  □

Next, we provide an example where the upper and lower bounds obtained in Theorems 8 and 9 are studied.

**Example** **5.** *Let $Y=(Y_{1},\ldots ,Y_{N})$ be an i.i.d Bernoulli(0.5) sequence and $X_{N}=\frac{1}{N}\sum_{i=1}^{N}Y_{i}$. Clearly, $X_{N}$ is a function of Y, |X|=N+1 and $\left|Y\right|=2^{N}$. Using ([1], (72)), we have*

(78) h0(PXY)=(a)H(Y|X)=∑i=1N(12)NNi,

*where (a) follows from ([1], (70)). Let the shared key size be N+1, by using Theorems 8 and 9, we have*

(79) ϵ+∑i=1N(12)NNi≤L(PXY,N+1,ϵ)≤log((N+2)(2N−N))+log(N+1),

*where *(75)* is used for the upper bound and $L_{3}\left(\epsilon ,P_{XY}\right)$ is used for the lower bound. Using ([1], (73)), we have*

(80) $$\begin{array}{c} \lim_{N\to \infty }\frac{h_{0}\left(P_{XY}\right)}{N}=\lim_{N\to \infty }\frac{H(Y|X)}{N}=h\left(0.5\right)=1, \end{array}$$

*where h(·) is the binary entropy function. Now, by using *(79)* and *(80)*, we obtain*

(81) $$\begin{array}{c} \lim_{N\to \infty }\frac{L(P_{XY},N+1,\epsilon )}{N}=1 \end{array}$$

*Bounds for Bounded Leakage Constraint*

Here, we assume that $X=(X_{1},X_{2})$, where $X=X_{1}\times X_{2}$, $1<|X_{1}\left|\leq \right|X_{2}|$, and $|X_{1}\left|\right|X_{2}\left|=|X\right|$. In the next theorem, we provide upper bounds for $L^{b}\left(P_{XY},M,\epsilon \right)$. We show that when the leakage is more than a threshold, we are able to communicate the message over the channel using a shared key size less than |X| and the receiver can decode the message without any loss.

**Theorem** **10.** *Let $\epsilon \geq H(X_{1})$ and the RVs $\left(X_{1},X_{2},Y\right)$ be distributed according to $P_{X_{1}X_{2}Y}$ and let the shared secret key size be $|X_{2}|$, i.e., $M=|X_{2}|$. Then, we have*

(82) $$\begin{array}{cc} \; & L^{b}(P_{XY},|X_{2}\left|,\epsilon \right)\leq \sum_{x\in X}\;\;H\left(Y|X=x\right)\;+H\left(X_{1}\right)\;+\;2+\;\left\lceil \log(\right|X_{2}\left|)\right\rceil , \end{array}$$

*if |Y| is finite, then*

(83) $$\begin{array}{cc} \; & L^{b}(P_{XY},|X_{2}\left|,\epsilon \right)\leq \left\lceil \log\left(|X|(|Y|-1)+1\right)\right\rceil +\left\lceil \log(\right|X_{2}\left|)\right\rceil +2+H\left(X_{1}\right), \end{array}$$

*and if $X=(X_{1},X_{2})$ is deterministic function of Y, we obtain*

(84) $$\begin{array}{cc} \; & L^{b}(P_{XY},|X_{2}\left|,\epsilon \right)\leq \left\lceil \log\left(|Y|-|X|+1\right)\right\rceil +\left\lceil \log(\right|X_{2}\left|)\right\rceil +2+H\left(X_{1}\right), \end{array}$$

*Furthermore, let $\epsilon \geq H(X_{2})$ and the shared secret key size be $|X_{1}|$. We have*

(85) $$\begin{array}{cc} \; & L^{b}(P_{XY},|X_{1}\left|,\epsilon \right)\leq \sum_{x\in X}\;\;H\left(Y|X=x\right)\;+H\left(X_{2}\right)\;+\;2+\;\left\lceil \log(\right|X_{1}\left|)\right\rceil , \end{array}$$

**Proof.**  The proof is similar to the proof of Theorem 8 and is provided in Appendix B.  □

**Remark** **14.** *As argued in ([1], Theorem 9), when the leakage is zero and X is a deterministic function of Y, if the shared key size is less than the size of X, i.e., M≤|X|, lossless variable-length codes do not exist. However, as is shown in Theorem 10, when the leakage is more than a threshold, for the key size less than |X|, such codes exist.*

**Remark** **15.** *The upper bounds *(82)* and *(85)* can be less than the upper bound found in ([1], Theorem 9), which is obtained under perfect privacy assumption. For instance, if $H\left(X_{1}\right)+2\leq \log(|X_{1}\left|\right)$, then *(82)* is less than ([1], (95)). The latter follows, since we have $H\left(X_{1}\right)+1+\left\lceil \log(\right|X_{2}\left|)\right\rceil \leq \log(|X_{1}\left|\right)+\left\lceil \log(|X|\right)-\log(|X_{1}\left|)\right\rceil -1\leq \left\lceil \log(\right|X_{1}\left|)\right\rceil +\left\lceil \log(|X|\right)-\log(|X_{1}\left|)\right\rceil -1\leq \left\lceil \log(|X|)\right\rceil ,$ where the last inequality follows by ⌈a⌉+⌈b⌉≤⌈a+b⌉+1.*

In the next theorem, we find lower bounds for $L^{b}\left(P_{XY},M,\epsilon \right)$.

**Theorem** **11.** *Let 0≤ϵ≤H(X) and the RVs $\left(X_{1},X_{2},Y\right)$ be distributed according to $P_{X_{1}X_{2}Y}$. For any shared secret key size M≥1, we have*

(86) $$\begin{array}{c} L^{b}\left(P_{XY},M,\epsilon \right)\geq H\left(Y|X\right)-H\left(X|Y\right). \end{array}$$

*Furthermore, if X is a deterministic function of Y, then we have*

(87) $$\begin{array}{c} L^{b}\left(P_{XY},M,\epsilon \right)\geq \log\left(\frac{1}{\max_{x}P_{X}\left(x\right)}\right). \end{array}$$

**Proof.**  The proof is provided in Appendix B.  □

*A General Approach for X*

Next, by using the same construction as in Theorem 10, we find upper bounds on $L^{b}\left(P_{XY},M,\epsilon \right)$ for any joint distribution $P_{XY}$. Next, we present a simple observation, which we call the *separation technique*.

**Observation** **1.** *(Separation technique) Any discrete RV X supported on X={1,…,|X|} with |X|>2 can be represented by two RVs $\left(X_{1},X_{2}\right)$, where $1<|X_{1}\left|<|X\right|$ and $1<|X_{2}\left|<|X\right|$.*

If |X| is not a prime number, then we can show *X* by $\left(X_{1},X_{2}\right)$ where $X=X_{1}\times X_{2}$, $1<|X_{1}\left|\leq \right|X_{2}|$, $|X_{1}\left|\right|X_{2}\left|=|X\right|$ and $P_{X}\left(x\right)=P_{X_{1},X_{2}}\left(x_{1},x_{2}\right)$. Furthermore, if |X| is a prime number, then we can show *X* by $\left(X_{1},X_{2}\right)$, where $|X_{1}\left|\right|X_{2}|=|X|+1$ and $P_{X_{1},X_{2}}\left(\right|X_{1}\left|,\right|X_{2}\left|\right)=0$. Let $S_{X}$ be all possible representations of *X*, where $X=(X_{1},X_{2})$. For a fixed ϵ, we define $S_{X}^{1\epsilon }≜\left\{\left(X_{1},X_{2}\right):\left(X_{1},X_{2}\right)\in S_{X},H\left(X_{1}\right)\leq \epsilon \right\}$ and $S_{X}^{2\epsilon }≜\left\{\left(X_{1},X_{2}\right):\left(X_{1},X_{2}\right)\in S_{X},H\left(X_{2}\right)\leq \epsilon \right\}$.

**Theorem** **12.** *For any ϵ≥0, pair of RVs (X,Y) distributed according to $P_{XY}$, and shared key size M≥α, if $S_{X}^{1\epsilon }\neq$, we have*

(88) $$\begin{array}{cc} \; & L^{b}\left(P_{XY},M,\epsilon \right)\leq \sum_{x\in X}\;\;H\left(Y|X=x\right)\;+\;2\;+\;\min_{\left(X_{1},X_{2}\right)\in S_{X}^{1\epsilon }}\left\{H\left(X_{1}\right)\;+\;\left\lceil \log(\right|X_{2}\left|)\right\rceil \right\}, \end{array}$$

*where $\alpha =|{arg min}_{X_{2}:\left(X_{1},X_{2}\right)\in S_{X}^{1\epsilon }}H\left(X_{1}\right)+\left\lceil \log(\right|X_{2}\left|)\right\rceil \left|<|X\right|$ and |S| denotes the size of the RV S. If $S_{X}^{2\epsilon }\neq$, for a shared key size M≥β, we have*

(89) $$\begin{array}{cc} \; & L^{b}\left(P_{XY},M,\epsilon \right)\leq \sum_{x\in X}\;\;H\left(Y|X=x\right)\;+\;2\;+\;\min_{\left(X_{1},X_{2}\right)\in S_{X}^{1\epsilon }}\left\{H\left(X_{2}\right)\;+\;\left\lceil \log(\right|X_{1}\left|)\right\rceil \right\}, \end{array}$$

*where $\alpha =|{arg min}_{X_{1}:\left(X_{1},X_{2}\right)\in S_{X}^{2\epsilon }}H\left(X_{1}\right)+\left\lceil \log(\right|X_{2}\left|)\right\rceil \left|<|X\right|$.*

**Proof.**  To prove (88), let $\left(X_{1},X_{2}\right)\in S_{X}^{1\epsilon }$. Then, by using Theorem 10, we have

(90) $$\begin{array}{cc} \; & L^{b}(P_{XY},|X_{2}\left|,\epsilon \right)\leq \sum_{x\in X}\;\;H\left(Y|X=x\right)\;+H\left(X_{1}\right)\;+\;2+\;\left\lceil \log(\right|X_{2}\left|)\right\rceil . \end{array}$$

Since (90) holds for any $\left(X_{1},X_{2}\right)\in S_{X}^{1\epsilon }$, we can take the minimum over $\left(X_{1},X_{2}\right)$, which results in (88). Furthermore, the key size is chosen as $|{arg min}_{X_{2}:\left(X_{1},X_{2}\right)\in S_{X}^{1\epsilon }}H\left(X_{1}\right)+\left\lceil \log(\right|X_{2}\left|)\right\rceil \left|<|X\right|$ and note that the first term $\sum_{x\in X}H\left(Y|X=x\right)$ remains the same, since it is a function of $P_{Y|X_{1}X_{2}}$, i.e., the distributions including the pair $\left(X_{1},X_{2}\right)$ do not change, since $P_{X_{1},X_{2}}\left(x_{1},x_{2}\right)=P_{X}\left(x\right)$. Using (85) and following the same approach, (89) is obtained.  □

**Remark** **16.** *The upper bounds *(88)* and *(89)* generalize *(82)* and *(85)*. The upper bounds *(82)* and *(85)* are obtained for a fixed $\left(X_{1},X_{2}\right)$. Thus, by taking the minimum over $\left(X_{1},X_{2}\right)\in S_{X}^{1\epsilon }$ and $\left(X_{1},X_{2}\right)\in S_{X}^{2\epsilon }$, we obtain tighter upper bounds.*

**Remark** **17.** *The same lower bounds as obtained in Theorem 11 can be used here, since the separation technique does not improve the bounds in *(86)* and *(87)*.*

**Remark** **18.** *Using Example 5, we can see that the bounds obtained in Theorems 10–13 can be asymptotically tight.*

Next, we provide an example to clarify the construction in Theorem 12 and compare the bounds with the perfect privacy case.

**Example** **6.** *Let the RV X∈{1,…,12} with distribution $P_{X}\left(x\right)=0.05,\;x\in \left\{1,\ldots ,10\right\}$ and $P_{X}\left(x\right)=0.475,\;x\in \left\{11,12\right\}$ be correlated with Y, where Y|X=x is a BSC(0.5). To calculate the bound in Theorem 12, one possible separation of X is $\left(X_{1},X_{2}\right)$, where the distributions of $X_{1}$ and $X_{2}$ are $P_{X_{1}}\left(x_{1}\right)=0.01,\;x\in \left\{1,\ldots ,5\right\}$, $P_{X_{1}}\left(x_{1}\right)=0.95,\;x_{1}=6$ and $P_{X_{2}}\left(x_{2}\right)=0.5,\;x\in \left\{1,2\right\}$. In this case, $H(X_{1})=0.4025$ and $\log(|X_{2}|)=1$. Using the bound *(82)*, for all ϵ≥0.4025 and key size M≥1, we have*

$$\begin{array}{cc} \; & L^{b}\left(P_{XY},M,\epsilon \right)\leq \;\;\;\sum_{x\in X}\;\;H\left(Y|X=x\right)\;+H\left(X_{1}\right)\;+\;2+\;\left\lceil \log(\right|X_{2}\left|)\right\rceil =15.45\;\mathrm{bits}, \end{array}$$

*By using the bound ([1], (95)), for the key size M≥12, we have*

$$\begin{array}{c} L^{b}\left(P_{XY},M,0\right)\leq 17\;\mathrm{bits}. \end{array}$$

*Note that, since |Y|=2, we can also use the following bound instead of *(88)

$$\begin{array}{cc} \; & L^{b}\left(P_{XY},M,\epsilon \right)\leq \left\lceil \log\left(|X|(|Y|-1)+1\right)\right\rceil +2\\ & +\min_{\left(X_{1},X_{2}\right)\in S_{X}^{1\epsilon }}\left\{\left\lceil \log(\right|X_{2}\left|)\right\rceil +H\left(X_{1}\right)\right\}. \end{array}$$

*By checking all possible separations, the minimum of $\left\lceil \log(\right|X_{2}\left|)\right\rceil +H\left(X_{1}\right)$ occurs at $|X_{1}|=6$, and $|X_{2}|=2$ where $X_{1}$ and $X_{2}$ have the distributions mentioned before. Thus, for all ϵ≥0.4025 and the key size M≥1, we have $L^{b}\left(P_{XY},M,\epsilon \right)\leq 7.4025\;\mathrm{bits}.$ By using the bound ([1], (96)), for the key size M≥12, we have $L^{b}\left(P_{XY},M,0\right)\leq 9\;\mathrm{bits}.$ We can see that by allowing a small amount of leakage with a shorter secret key size, we are able to send shorter codewords.*

In the following, we study the bounds obtained in Theorem 12 considering a perfect privacy scenario and we show that the previous bounds can be improved by using the separation technique.

*Perfect Privacy (* ϵ=0 *)*

In this part, we let ϵ=0 and consider the problem as in [1]. We show that when $X=(X_{1},X_{2})$, where $X_{2}$ is a deterministic function of $X_{1}$, the upper bounds on $L^{b}\left(P_{XY},M,0\right)$ can be improved by using a shorter shared key size, i.e., M<|X|. In the next theorem, we provide upper bounds on $L^{b}\left(P_{XY},M,0\right)$.

**Theorem** **13.** *Let $X=(X_{1},X_{2})$, where $X_{2}=f\left(X_{1}\right)$, and the shared key size be $|X_{1}|$. We have*

(91) $$\begin{array}{cc} \; & L^{b}(P_{XY},|X_{1}\left|,0\right)\leq \sum_{x_{1}\in X_{1}}\;\;H\left(Y|X_{1}=x_{1}\right)\;+\;1\;+\;\left\lceil \log(\right|X_{1}\left|)\right\rceil , \end{array}$$

*if |Y| is finite, then*

(92) $$\begin{array}{cc} \; & L^{b}(P_{XY},|X_{1}\left|,0\right)\leq \left\lceil \log\left(|X|(|Y|-1)+1\right)\right\rceil +\left\lceil \log(\right|X_{1}\left|)\right\rceil +1, \end{array}$$

*and if $X=(X_{1},X_{2})$ is deterministic function of Y, we obtain*

(93) $$\begin{array}{c} L^{b}(P_{XY},|X_{1}\left|,0\right)\leq \left\lceil \log\left(|Y|-|X|+1\right)\right\rceil \;+\;\left\lceil \log(\right|X_{1}\left|)\right\rceil \;+\;1. \end{array}$$

**Proof.**  The proof is provided in Appendix B.  □

**Remark** **19.** *Clearly, when $X=(X_{1},X_{2})$ with $X_{2}=f\left(X_{1}\right)$ the upper bounds *(91)*, *(92)* and *(93)* improve the bounds in ([1], Theorem 8).*

*Special Case: $X=(X_{1},X_{2})$ and $X_{2}=f\left(X_{1}\right)$*

In this part, we use similar approaches as used in Theorems 12 and 13 to find upper bounds. We encode $X_{1}$ using one-time-pad coding and the same *U* as in Theorem 13. As shown in Theorem 13, $X_{1}$ is sufficient to decode *Y*, since $X_{2}=f\left(X_{1}\right)$; however, in this scheme, we do not have the possibility that we are allowed to leak about *X*. Similarly to Theorem 13, we obtain

$$\begin{array}{c} L^{b}(P_{XY},|X_{1}\left|,\epsilon \right)\leq \sum_{x\in X}\;\;H\left(Y|X=x\right)\;+\;1\;+\;\left\lceil \log(\right|X_{1}\left|)\right\rceil \\ =\sum_{x_{1}\in X_{1}}\;\;H\left(Y|X_{1}=x_{1}\right)\;+\;1\;+\;\left\lceil \log(\right|X_{1}\left|)\right\rceil . \end{array}$$

Next, we use the separation technique as used in Theorem 12. For any ϵ≥0, if a separation exist such that $\epsilon \geq H(X_{1}^{\prime })$, where $X_{1}=\left(X_{1}^{\prime },X_{1}^{\prime \prime }\right)$, we obtain

$$\begin{array}{cc} \; & L^{b}(P_{XY},|X^{\prime \prime }\left|,\epsilon \right)\leq \sum_{x_{1}\in X_{1}}H\left(Y|X_{1}=x_{1}\right)+H\left(X_{1}^{\prime }\right)+2+\left\lceil \log(\right|X_{1}^{\prime \prime }\left|)\right\rceil . \end{array}$$

Furthermore, we can take the minimum over all possible separations to improve the upper bound. Let $S_{X_{1}}^{\epsilon }$ be all possible separations, where $\epsilon \geq H(X_{1}^{\prime })$. We have

$$\begin{array}{cc} \; & L^{b}\left(P_{XY},\alpha ,\epsilon \right)\leq \sum_{x_{1}\in X_{1}}\;\;H\left(Y|X_{1}=x_{1}\;\right)\;+\;2\;+\;\min_{\left(X_{1}^{\prime },X_{1}^{\prime \prime }\right)\in S_{X_{1}}^{\epsilon }}\;\;\left\{\;H\left(X_{1}^{\prime }\right)\;+\;\left\lceil \log(\right|X_{1}^{\prime \prime }\left|)\right\rceil \;\right\}, \end{array}$$

where $\alpha =|{arg min}_{X_{1}^{\prime \prime }:\left(X_{1}^{\prime },X_{1}^{\prime \prime }\right)\in S_{X_{1}}^{\epsilon }}H\left(X_{1}^{\prime }\right)+\left\lceil \log(\right|X_{1}^{\prime \prime }\left|)\right\rceil \left|<\right|X_{1}|$.

We can see that the upper bound on $L^{b}(P_{XY},|X^{\prime \prime }\left|,\epsilon \right)$ can be less than the bound in (91). For instance, let $H\left(X_{1}^{\prime }\right)+2\leq \log(|X_{1}^{\prime }\left|\right)$. Using the same arguments as in Remark 15, we have $H\left(X_{1}^{\prime }\right)+1+\left\lceil \log(\right|X_{1}^{\prime \prime }\left|)\right\rceil \leq \left\lceil \log(\right|X_{1}\left|)\right\rceil$. Hence, if the leakage is more than a certain threshold, this can help us to improve the upper bound and send shorter codewords compared to the perfect privacy case. Moreover, it helps us to use a shorter shared secret key size.

## 5. Applications: Cache-Aided Networks

**Figure 7:**
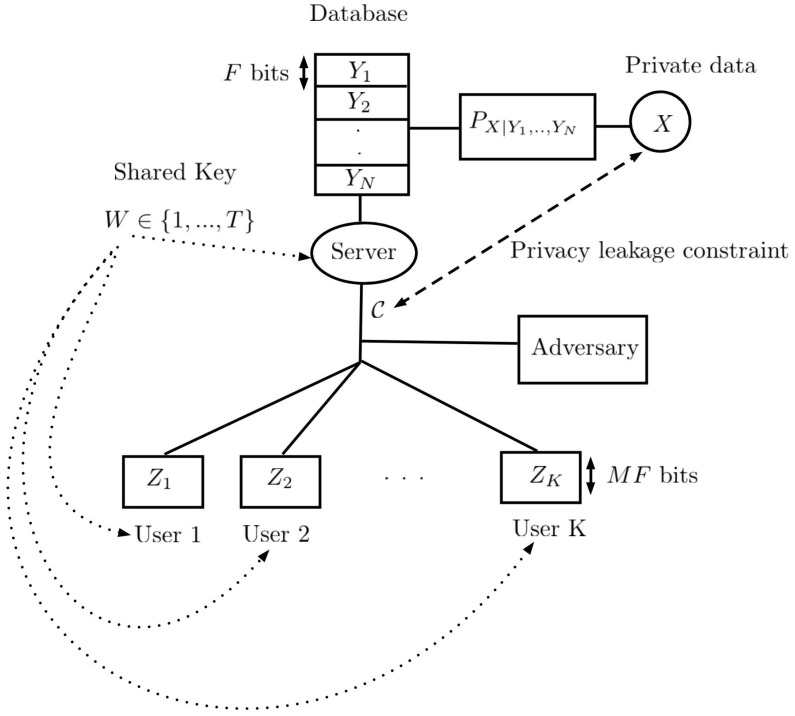
A server wants to send a response over a shared link to the satisfy users’ demands, but since the database is correlated with the private data, the existing schemes are not applicable. In the delivery phase, we hide the information about *X* using one-time-pad coding and send the rest of response using the functional representation lemma (FRL).

In this part, we present an application where the results of this paper can be used. The complete study of the application can be found in [49]. To do so, let us consider the scenario illustrated in Figure 7. A server has access to a database consisting of *N* files $Y_{1},\ldots ,Y_{N}$, where each file, of size *F* bits, is sampled from the joint distribution $P_{XY_{1}\cdot Y_{N}}$. The random variable *X* denotes the private latent variable. We assume that the server knows the realization of the private variable *X* as well. The server is connected to *K* users over a shared link, where user *i* has access to a local cache memory of size MF bits. Furthermore, we assume that the server and the users have access to a shared secret key denoted by *W*, of size *T*. The system works in two phases: the placement and delivery phases, respectively [50]. In the placement phase, the server fills the local caches using the database. Let $Z_{k}$ denote the content of the local cache memory of user *k*, k∈[K]≜{1,…,K} after the placement phase. In the delivery phase, first the users send their demands to the server, where $d_{k}\in \left[N\right]$ denotes the demand of user *k*. The server sends a response, denoted by C, over the shared link to satisfy all the demands, simultaneously. We assume that an adversary has access to the shared link as well, and uses C to extract information about *X*. However, the adversary does not have access to the local cache contents or the secret key. Since the files in the database are all correlated with the private latent variable *X*, the coded caching and delivery techniques introduced in [50] do not satisfy the privacy requirement. The goal of the cache-aided private delivery problem is to find a response C with minimum possible average length that satisfies a certain privacy constraint and the zero-error decodability constraint of the users. Here, we consider the worst-case demand combinations $d=(d_{1},\ldots ,d_{K})$ to construct C. This expectation is taken for the randomness in the database. In this study, we first consider a perfect privacy constraint, i.e., we require C to be independent of *X*. Let ${\hat{Y}}_{d_{k}}$ denote the decoded message of user *k* using *W*, C, and $Z_{k}$. User *k* should be able to recover $Y_{d_{k}}$ reliably, i.e., $P\left\{{\hat{Y}}_{d_{k}}\neq Y_{d_{k}}\right\}=0$, ∀k∈[K]. We remark that we have a cache-aided variable-length compression problem with a privacy constraint. Hence, to solve this problem and design the code C, we utilized techniques used in privacy mechanisms, data compression, and cache design and coded delivery problems, and combined them to build such a code. In particular, we used the data compression techniques employed in this paper and caching design techniques in [50]. Finally, we extended the results for correlated *X* and C, i.e., when non-zero leakage is allowed. To do so, we used the results of this paper for correlated *X* and C, i.e., the non-perfect privacy part. For more details, see [49].

## 6. Experiment

**Figure 8:**
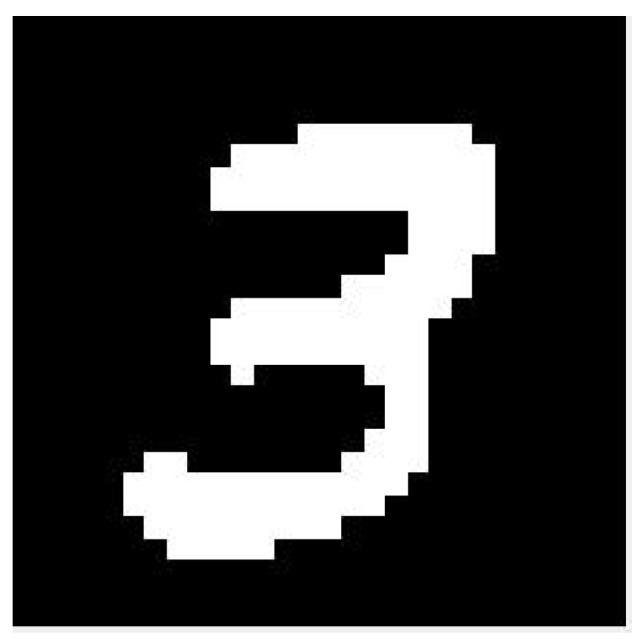
Quantized image of digit 3 with resolution 28×28.

In this section, we provide an experiment to evaluate the bounds obtained in Theorem 4. To do so, we used the MNIST dataset, which contains 60,000 images illustrating handwritten digits from 0 to 9. Let the RV *S* (information source) denote the images in the dataset, i.e., |S|= 60,000. Let *Y* represent the useful data that is a feature extracted from the dataset. In this example, the useful data are defined as a “quantized histogram”. To determine the quantized histogram of the dataset, we first converted the images into black and white by quantizing them. The histogram of any image in the dataset is then computed as the ratio of the number of white pixels to the total number of pixels. Consequently, the histogram of any image can be represented by a value within the interval [0,1]. For quantizing the histogram of any image, we used the following intervals: Interval 1 =[0,0.1), Interval 2 =[0.1,0.15), Interval 3 =[0.15,0.2), Interval 4 =[0.2,0.25), Interval 5 =[0.25,0.3), Interval 6 =[0.3,0.35) and Interval 7 =[0.35,1]. Each image in the dataset was therefore labeled with a number between 1 and 7, indicating the interval to which its histogram belongs. Let *Y* denote the interval sample *S* belongs to. Clearly, the labels of the intervals are deterministic functions of the information source *S*. For instance, in Figure 8, the number of white pixels is 130, hence the quantized histogram is equal to 0.1658, which corresponds to the third interval.

Let the RV *Z* denote the label of each image that is a number from 0 to 9, indicating the digit which the image represents, i.e., Z∈{0,1,…,9}. Clearly, *Z* is a deterministic function of *S*, i.e., Z=f(S). The private attribute *X* is defined as the presence of, digit 0 which is a binary RV, i.e., X∈{0,1} and $X=\left\{\begin{array}{c} 1\;\mathrm{if}\;Z=0,\\ 0\;\mathrm{if}\;Z=1 \end{array}\right.$. Here, we use an empirical distribution for *Z* and it can be seen that *X* is a deterministic function of *Z*, which yields the Markov chain X−Z−Y. Using this Markov chain, the kernel $P_{X|Y}$ can be obtained as $P_{X|Z}P_{Z|Y}$, where

$$\begin{array}{c} P_{X|Z}=\left[\begin{array}{ccccccccc} 1 & 0 & 0 & 0 & 0 & 0 & 0 & 0 & 0\\ 0 & 1 & 1 & 1 & 1 & 1 & 1 & 1 & 1 \end{array}\right], \end{array}$$

and $P_{Z|Y}$ can be found from the dataset. We used an empirical distribution to find the kernel $P_{Y|Z}$, e.g., $P_{Y=1|Z=1}$ is the number of images illustrating digit 1, which corresponds to label 1 (their quantized histograms belong to the first interval) divided by the total number of images with digit 1. By using $P_{Y|Z}$ derived in (94) and multiplying it by $P_{Z|X}$, we obtain $P_{X|Y}$ as in (95).

(94) $$\begin{array}{c} P_{Z|Y}=\left[\begin{array}{ccccccc} 0.0064 & 0.0441 & 0.1445 & 0.3070 & 0.4678 & 0.5968 & 0.7500\\ 0.4922 & 0.0497 & 0.0037 & 0.0004 & 0 & 0 & 0\\ 0.0262 & 0.0854 & 0.1440 & 0.1599 & 0.1044 & 0.1129 & 0\\ 0.0416 & 0.1017 & 0.1339 & 0.1265 & 0.0922 & 0.0323 & 0\\ 0.0876 & 0.1330 & 0.0796 & 0.0332 & 0.0111 & 0 & 0\\ 0.0712 & 0.1058 & 0.0907 & 0.0676 & 0.0478 & 0.0161 & 0\\ 0.0479 & 0.1086 & 0.1181 & 0.0973 & 0.0711 & 0.0645 & 0\\ 0.1330 & 0.1447 & 0.0635 & 0.0201 & 0.0078 & 0 & 0\\ 0.0162 & 0.0872 & 0.1409 & 0.1523 & 0.1700 & 0.1774 & 0.2500\\ 0.0778 & 0.1396 & 0.0812 & 0.0357 & 0.0278 & 0 & 0 \end{array}\right], \end{array}$$

(95) $$\begin{array}{c} P_{X|Y}=\left[\begin{array}{ccccccc} 0.0064 & 0.0441 & 0.1445 & 0.3070 & 0.4678 & 0.5968 & 0.7500\\ 0.9936 & 0.9559 & 0.8555 & 0.6930 & 0.5322 & 0.4032 & 0.2500 \end{array}\right]. \end{array}$$

Moreover, the distribution of the task *Y* is given as in Table 1.

The goal was to compress *Y* and send it to a user, and we considered a perfect privacy case where the privacy leakage was zero. Since *X* is a deterministic function of *Y*, we have $h_{0}\left(P_{XY}\right)=g_{0}\left(P_{XY}\right)$ and $P_{XY}\in {\hat{P}}_{XY}$. Hence, using the linear program in [15], we found *U* that achieved $g_{0}(P_{XY}$, and $K(P_{XY})$ was obtained. Using Theorem 4, the average length of C was 2.88 bits; however, using the method in [1], 4.16 bits were required to send the message C over the channel.

## 7. Conclusions

We have studied a compression problem with a privacy constraint in two scenarios, where the information delivered over the channel is either independent of *X* or is correlated with *X*, satisfying a bounded leakage constraint. Considering the perfect privacy constraint, we proposed new upper bounds using a two-part construction coding that benefits from the solution of $g_{0}\left(P_{XY}\right)=h_{0}\left(P_{XY}\right)$ to encode the second part of the code. For the first part of the code, we hide the private data using one-time pad coding. Furthermore, in case of |Y|≥|X|, we proposed a new achievable scheme. We have shown that the new upper bounds can improve the existing ones. Moreover, we strengthened the bounds using the greedy entropy-based algorithm. Considering the second scenario, we obtained upper and lower bounds and showed that the upper bounds can be asymptotically tight. To achieve the upper bounds, we used two-part construction coding. Furthermore, in the case of perfect privacy, we showed that, by using the separation technique, the previous upper bounds can be improved.

## Figures and Tables

**Table 1:** Marginal distribution of the useful data *Y*.

	y=1	y=2	y=3	y=4	y=5
Pr(Y=y)	0.1851	0.4069	0.2981	0.0936	0.0151
	y=6	y=7			
Pr(Y=y)	0.0010	0.0002			

**Figure A1:**
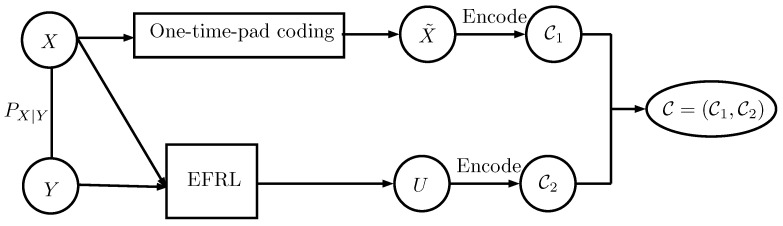
We use a two-part construction coding strategy to send codewords over the channels. As discussed in Theorem 8, on the receiver side using C and the shared key, we decode *X*, and using *X*, the shared key, and C, we decode *Y*. The leakage from *X* to C equals ϵ.

**Figure A2:**
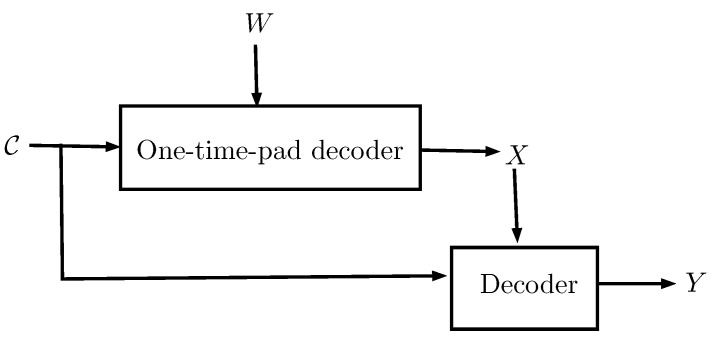
On the receiver side, we first decode *X* using the shared key *W*, then by using the fact that *U* is produced by EFRL, we have H(Y|X,U)=0. Thus, we can decode *Y* based on *X* and *U*.

## Data Availability

The data presented in this study are openly available in https://doi.org/10.1109/WIFS58808.2023.10374604.

## Appendix A

### Appendix A.1. Proof of Theorem 4

Let $U\in U^{1}$. In the following, we expand H(Y,U|X=x) using the chain rule:

(A1) $$\begin{array}{cc} H(Y,U|X=x) & =H(U|X=x)+H(Y|U,X=x)\\ & \overset{\left(a\right)}{=}H\left(U\right), \end{array}$$

where, in (a), we used

$$\begin{array}{c} I(X;U)=0\to H(U)=H(U|X)=H(U|X=x), \end{array}$$

and

$$\begin{array}{c} H(Y|X,U)=0\to H(Y|U,X=x)=0. \end{array}$$

Furthermore,

(A2) $$\begin{array}{cc} H(Y,U|X=x) & =H(Y|X=x)+H(U|Y,X=x)\\ & =\;\;H\left(Y|X\;=\;x\right)\;+\;\;\;\sum_{y}\;\;P_{y|x}H\left(U|Y\;=\;y,\;X\;=\;x\right)\\ & \overset{\left(b\right)}{=}\;\;H\left(Y|X\;=\;x\right)\;+\;\sum_{y}\;\;P_{y|x}H\left(U|Y\;=\;y\right), \end{array}$$

where (b) follows from the Markov chain X−Y−U. Combining (A1) and (A2), for any x∈X, we have

(A3) $$\begin{array}{c} H\left(U\right)=H\left(Y|X\;=\;x\right)\;+\;\sum_{y}\;P_{y|x}H\left(U|Y\;=\;y\right). \end{array}$$

Furthermore, by using Lemma 4, *U* is an optimizer of both $g_{0}\left(P_{XY}\right)$ and $h_{0}\left(P_{XY}\right)$. Thus,

(A4) $$\begin{array}{c} H\left(U\right)\;=\;H\left(Y|X\right)\;+\;U\left(U|Y\right)\;=\;H\left(Y|X\right)\;+\;\;\;\sum_{y}\;P_{y}H\left(U|Y=y\right) \end{array}$$

Combining (A4) and (A3), we have

(A5) $$\begin{array}{c} H\left(Y|X\right)\;+\;\;\;\sum_{y}\;P_{y}H\left(U|Y=y\right)\;=\;H\left(Y|X\;=\;x\right)\;+\;\sum_{y}\;P_{y|x}H\left(U|Y\;=\;y\right) \end{array}$$

Thus, for any x∈X

(A6) $$\begin{array}{c} \sum_{y}\left(P_{y|x}-P_{y}\right)H\left(U|Y\;=\;y\right)=H\left(Y|X\right)-H\left(Y|X=x\right). \end{array}$$

For any optimizer $U\in U^{1}$, (A6) holds; hence, if we take the maximum over H(U|Y=y) and by letting $a_{i}=H\left(U|Y=y_{i}\right)$, we obtain

(A7) $$\begin{array}{c} H\left(U\right)\leq \max H\left(U\right)=H\left(Y|X\right)+\max\sum_{i}P_{y_{i}}a_{i} \end{array}$$

subject to

(A8) $$\begin{array}{c} A_{XY}a=b_{XY},\;a\geq 0. \end{array}$$

To prove (49), note that, since we consider all optimizers of $g_{0}\left(P_{XY}\right)$, there exists at least one $U^{*}$ that satisfies $|U^{*}|\leq \mathrm{null}\left(P_{X|Y}\right)+1$, which results $H\left(U^{*}\right)\leq \log\left(\mathrm{null}\left(P_{X|Y}\right)+1\right)$. Since (A6) must hold for any optimizer, $U^{*}$ satisfies the inequality in (49). Moreover, (50) follows from Lemma 6 and the constraint we added to have $H\left(U\right)\leq \log(\mathrm{null}\left(P_{X|Y}\right)+1)$. To prove the last statement, note that when Y=f(X), using the key equation in (36), the upper bound on $h_{0}\left(P_{XY}\right)$ equals zero, which results in $h_{0}\left(P_{XY}\right)=g_{0}\left(P_{XY}\right)=0$. Furthermore, as shown in ([1], Theorem 5), when Y≠f(X), we have $h_{0}\left(P_{XY}\right)>0$; thus, the system of linear equations in (A6) has at least one solution. However, due to $\mathrm{rank}(A_{XY})=|Y|$, it has a unique solution, since the number of independent equations is at least the number of variables. Finally, to prove the lower bound in (47), note that for any *U* that satisfies (33), (34) and (35), (A6) holds. By taking the minimum over $H\left(U\right)=H\left(Y|X\right)+\sum_{i}P_{y_{i}}H\left(U|y_{i}\right)$ subject to (A6). Moreover, the inequality holds for $U^{*}$ that achieves the minimum entropy.

### Appendix A.2. Proof of Theorem 5

Let *W* be the shared secret key with key size M=|X|, which is uniformly distributed over {1,…,M}={1,…,|X|} and independent of (X,Y). As shown in Figure 4, first, the private data *X* are encoded using the shared secret key ([2], Lemma 1). Thus, we have

$$\begin{array}{c} \tilde{X}=X+W\;\mathrm{mod}\;\left|X\right|. \end{array}$$

Next, we show that $\tilde{X}$ has a uniform distribution over {1,…,|X|} and $I(X;\tilde{X})=0$. We have

(A9) $$\begin{array}{c} H\left(\tilde{X}|X\right)\;=\;H\left(X\;+\;W|X\right)\;=\;H\left(W|X\right)\;=\;H\left(W\right)\;=\;\log\left(|X|\right). \end{array}$$

Furthermore, $H\left(\tilde{X}|X\right)\leq H\left(\tilde{X}\right)$, and combining it with (A9), we obtain $H\left(\tilde{X}|X\right)=H\left(\tilde{X}\right)=\log\left(|X|\right)$. For encoding $\tilde{X}$, we use ⌈log(|X|)⌉ bits. Let $C_{1}$ denote the encoded $\tilde{X}$. Next, by using $P_{XY}\in {\hat{P}}_{XY}$, let $U^{*}\in U^{1}$ be an optimizer of $h_{0}\left(P_{XY}\right)=g_{0}\left(P_{XY}\right)$ that achieves $K(P_{XY})$, i.e., has the minimum entropy. We encode $U^{*}$ using any lossless code, which uses at most $H\left(U^{*}\right)+1=K\left(P_{XY}\right)+1$ bits and let $C_{2}$ be the encoded message $U^{*}$. We send $C=(C_{1},C_{2})$ over the channel. Thus, (52) can be obtained. Note that, as shown in Figure 5, on the decoder side, we first decode *X* using *W*, by adding |X|−W to $\tilde{X}$, then, *Y* is decoded using the fact that $U^{*}$ satisfies $H(Y|U^{*},X)=0$. Next, we show that if *W* is independent of $\left(X,U^{*}\right)$, we obtain I(C;X)=0. We have

$$\begin{array}{cc} I(C;X) & =I\left(C_{1},C_{2};X\right)=I\left(U,\tilde{X};X\right)\\ & =I\left(U^{*};X\right)+I\left(\tilde{X};X|U\right)=I\left(\tilde{X};X|U^{*}\right)\\ & \overset{\left(a\right)}{=}H\left(\tilde{X}|U^{*}\right)-H\left(\tilde{X}|X,U^{*}\right)\\ & =H\left(\tilde{X}|U^{*}\right)-H\left(X+W|X,U^{*}\right)\\ & \overset{\left(b\right)}{=}H\left(\tilde{X}|U^{*}\right)-H\left(W\right)\\ & \overset{\left(c\right)}{=}H\left(\tilde{X}\right)-H\left(W\right)\\ & =\log(|X|)\;-\;\log(|X|)\\ & =0 \end{array}$$

where (a) follows from $I(U^{*};X)=0$; (b) follows, since *W* is independent of $\left(X,U^{*}\right)$; and (c) from the independence of $U^{*}$ and $\tilde{X}$. The latter follows, since we have

$$\begin{array}{cc} 0\leq I(\tilde{X};X|U^{*}) & =H\left(\tilde{X}|U^{*}\right)-H\left(W\right)\\ & \overset{\left(i\right)}{=}H\left(\tilde{X}|U^{*}\right)-H\left(\tilde{X}\right)\\ & \leq 0. \end{array}$$

Thus, $\tilde{X}$ and $U^{*}$ are independent. Step (i) above follows from the fact that *W* and $\tilde{X}$ are uniformly distributed over {1,…,|X|}, i.e., $H\left(W\right)=H\left(\tilde{X}\right)$. As a summary, if we choose *W* independent of $\left(X,U^{*}\right)$, the leakage to the adversary is zero. Thus, (52) is achievable. The upper bounds (53) and (54) can be obtained using the same coding and chain of inequalities stated in Theorem 4. The upper bounds in (56) and (57) were derived in ([1], Theorem 8). The upper bound in (55) can be obtained using the same coding. Note that the inequality between the upper bounds follows from the fact that the RV *U* that achieves the upper bound in (56) does not necessarily minimize the entropy H(U); however, it satisfies the constraints I(X;U)=0 and H(Y|X,U)=0. Finally, to achieve (58), let the shared key *W* be independent of (X,Y) and have a uniform distribution. We construct $\tilde{Y}$ using one-time pad coding. We have

$$\begin{array}{c} \tilde{Y}=Y+W\;\mathrm{mod}\;\left|Y\right|. \end{array}$$

Then, $\tilde{Y}$ is encoded using any lossless code which uses at most ⌈log(|Y|)⌉ bits. The latter follows, since $\tilde{Y}$ has a uniform distribution. It only remains to show that $I(\tilde{Y};X)=0$. We have

$$\begin{array}{cc} I(\tilde{Y};X,Y) & =I(Y+W;X,Y)\\ & =H(Y+W)-H(Y+W|X,Y)\\ & =\log(|Y|)-H(W|X,Y)\\ & =\log(|Y|)-H(W)\\ & =\log(|Y|)-\log(|Y|)\\ & =0. \end{array}$$

Hence, $I(\tilde{Y};X)=0$. Furthermore, on the decoder side, we can decode *Y* using *W*.

## Appendix B

### Appendix B.1. Proof of Lemma 7

First we obtain $L_{3}\left(\epsilon ,P_{XY}\right)$. Using the key equation ([19], (7)) with assignments X←X and Y←(Y,W), for any RVs *X*, *Y*, *W*, and *U*, we have

(A10) $$\begin{array}{cc} I(U;Y,W) & =I(U;X)+H(Y,W|X)\\ & -H(Y,W|X,U)-I(X;U|Y,W). \end{array}$$

Using (A10), if *U* satisfies I(U;X)=ϵ and H(Y|U,X,W)=0 and *W* is independent of (X,Y), we have

$$\begin{array}{cc} H(U) & \geq I(U;Y,W)\\ & =I(U;X)+H(Y|X)+H(W|X,Y)\\ & -H(W|X,U)-H(Y|X,U,W)-H(X|Y,W)\\ & +H(X|W,Y,U)\\ & \overset{\left(a\right)}{=}\epsilon +H\left(Y|X\right)+H\left(W\right)-H\left(W|X,U\right)\\ & -H(X|Y)+H(X|Y,U,W)\\ & \overset{\left(b\right)}{\geq }\epsilon +H\left(Y|X\right)-H\left(X|Y\right), \end{array}$$

where in (a), we used I(X;U)=ϵ independently of *W* and (X,Y), and H(Y|X,U,W)=0. Step (b) follows from the non-negativity of H(W)−H(W|X,U)=I(W;X,U) and H(X|Y,U,W).

To prove $L_{2}\left(\epsilon ,P_{XY}\right)$, note that we have

(A11) $$\begin{array}{c} H\left(U\right)\geq \min_{x}H\left(U|X=x\right)+\epsilon , \end{array}$$

since

$$\begin{array}{cc} H\left(U\right)\;-\;\min_{x}H\left(U|X\;\;=\;x\right)\; & \geq \;H\left(U\right)\;-\;\sum_{x}\;P_{X}\left(x\right)H\left(U|X\;\;=\;x\right)\\ & =\epsilon . \end{array}$$

Furthermore,

(A12)H(U|X=x)≥H(U|W,X=x)≥(a)H(U|W,X=x)+H(Y|U,W,X=x)=H(U,Y|W,X=x)=H(Y|W,X=x)+H(U|Y,W,X=x)(A13)≥(b)H(Y|W,X=x)(A14)=H(Y|X=x),

where (a) used the fact that H(Y|U,W,X=x)=0, since H(Y|U,W,X)=0. (b) follows, since *W* is independent of *X* and *Y*. Taking the minimum on both sides, we obtain $\min_{x}H\left(U|X=x\right)\geq \min_{x}H\left(Y|X=x\right)$. Combining this with (A11), we obtain $L_{2}\left(\epsilon ,P_{XY}\right)$. To obtain $L_{1}\left(\epsilon ,P_{XY}\right)$, we take the average from both sides of (A12) and (A14). This gives us

$$\begin{array}{c} H(U)\geq H(U|X)\geq H(Y|X), \end{array}$$

which completes the proof.

### Appendix B.2. Proof of Theorem 8

To prove (73), we use the two-part construction coding scheme as used in [1]. As shown in Figure A1, first the private data *X* are encoded by one-time-pad coding ([2], Lemma 1), which uses ⌈log(|X|)⌉ bits. Next, we produce *U* based on EFRL ([19], Lemma 3) and encode it using any traditional code which uses at most H(U)+1 bits. By using the upper bound on H(U) found in ([19], Lemma 5), we obtain

$$\begin{array}{cc} \; & L(P_{XY},|X|,\epsilon )\leq \\ & \;\;\;\;\;\;\;\;\;\sum_{x\in X}\;\;H\left(Y|X=x\right)\;+\;\epsilon \;+\;h\left(\alpha \right)\;+\;1+\;\left\lceil \log(|X|)\right\rceil . \end{array}$$

For proving (74) and (75), we use the same coding and note that when |Y| is finite, we use the bound $\left|U\right|\leq \left[|X|(|Y|-1)+1\right]\left[|X|+1\right]$, which results in (74). Furthermore, when *X* is a deterministic function of *Y*, we can use the bound $\left|U\right|\leq \left[|Y|-|X|+1\right]\left[|X|+1\right]$ that leads to (75). Moreover, for the leakage constraint, we note that the randomness of the one-time-pad coding is independent of *X* and the output of the EFRL. Let *Z* be the compressed output of the EFRL and $\tilde{X}$ be the output of the one-time-pad coding. Then, we have

$$\begin{array}{cc} I(X;\tilde{X},Z) & =I\left(X;\tilde{X}\right)+I\left(X;Z|\tilde{X}\right)\\ & =I(X;Z)\\ & =\epsilon , \end{array}$$

where we used the independency between $\tilde{X}$ and (Z,X). Moreover, as shown in Figure A2, on the receiver side, we first decode *X* using the shared key *W*, then by using the fact that *U* is produced by EFRL, we can decode *Y* based on *X* and *U*.

### Appendix B.3. Proof of Theorem 9

Let $\tilde{U}=C\left(Y,W\right)$ be the output of the encoder. Since the code is ϵ-private, it satisfies I(U;X)=ϵ. Due to recoverability, we can recover the useful data, and this also satisfies H(Y|U,X,W)=0. Thus, by using Lemma 7, we have

$$\begin{array}{c} H\left(\tilde{U}\right)\geq \max\left\{L_{1}\left(\epsilon ,P_{XY}\right),L_{2}\left(\epsilon ,P_{XY}\right),L_{3}\left(\epsilon ,P_{XY}\right)\right\}, \end{array}$$

which results in (76). The proof of (77) is similar to ([1], Theorem 9). For any $\tilde{u}\in \xi$, we have

$$\begin{array}{cc} P_{\tilde{U}}\left(\tilde{u}\right) & =\sum_{x}P_{X}\left(x\right)P_{\tilde{U}|X}\left(\tilde{u}|x\right)\\ & \leq \max_{x}P_{X}\left(x\right)\sum_{x}P_{\tilde{U}|X}\left(\tilde{u}|x\right)\\ & =\max_{x}P_{X}\left(x\right)\sum_{x}\sum_{w}P_{\tilde{U}|WX}\left(\tilde{u}|w,x\right)P_{W}\left(w\right)\\ & =\max_{x}P_{X}\left(x\right)\sum_{w}P_{W}\left(w\right)\sum_{x}P_{\tilde{U}|WX}\left(\tilde{u}|w,x\right)\\ & \overset{\left(a\right)}{\leq }\max_{x}P_{X}\left(x\right)\sum_{w}P_{W}\left(w\right)=\max_{x}P_{X}\left(x\right), \end{array}$$

where (a) follows from the lossless condition, since, as argued in ([1], Theorem 9), for a lossless code $P_{\tilde{U}|WX}\left(\tilde{u}|w,x\right)>0$ for at most one x∈X. Hence, we have

$$\begin{array}{c} H\left(\tilde{U}\right)\geq \log\left(\frac{1}{\max_{x}P_{X}\left(x\right)}\right). \end{array}$$

### Appendix B.4. Proof of Theorem 10

To prove (82), $X_{2}$ is encoded by one-time-pad coding ([2], Lemma 1), which uses $\left\lceil \log(\right|X_{2}\left|)\right\rceil$ bits. Let the output of the one-time-pad coding be ${\tilde{X}}_{2}$. Next, we encode $X_{1}$ using any traditional code, which uses at most $H(X_{1})+1$ bits. Next, we produce *U* based on FRL ([1], Lemma 1) with assignments $X\leftarrow (X_{1},X_{2})$ and Y←Y, and encode it using any traditional code, which uses at most H(U)+1 bits. We send the encoded $X_{1}$, ${\tilde{X}}_{2}$ and *U* over the channel. Since *U* satisfies $I(U;X_{1},X_{2})=0$ and $H(Y|U,X_{1},X_{2})=0$ by using the upper bound on H(U) found in ([1], Lemma 2), we obtain

$$\begin{array}{c} H\left(U\right)\leq \sum_{x}H\left(Y|X=x\right). \end{array}$$

Finally, we obtain

$$\begin{array}{cc} \; & L^{b}(P_{XY},|X_{2}\left|,\epsilon \right)\leq \\ & \;\;\;\;\;\sum_{x\in X}\;\;H\left(Y|X=x\right)\;+H\left(X_{1}\right)\;+\;2+\;\left\lceil \log(\right|X_{2}\left|)\right\rceil . \end{array}$$

Note that, on the receiver side, using the shared key and ${\tilde{X}}_{2}$ we can decode $X_{2}$, and by using $X_{1}$, $X_{2}$ and *U* we can decode *Y* without loss. For the leakage constraint, we note that the one-time-pad coding can be used such that ${\tilde{X}}_{2}$ is independent of $\left(X_{1},X_{2},U\right)$. Thus, we have

$$\begin{array}{cc} \; & I(X_{1},{\tilde{X}}_{2},U;X_{1},X_{2})=\\ & H\left(X_{1}\right)\;+\;H\left({\tilde{X}}_{2}\right)\;+\;H\left(U\right)\;-\;H\left(X_{1}|X_{1},X_{2}\right)\;-\;H\left({\tilde{X}}_{2}|X_{1},X_{2}\right)\\ & -H\left(U|X_{1},X_{2},{\tilde{X}}_{2}\right)\overset{\left(a\right)}{=}H\left(X_{1}\right)\leq \epsilon , \end{array}$$

where (a) follows from the independency between ${\tilde{X}}_{2}$ and $\left(U,X_{1},X_{2}\right)$, and independency between *U* and $\left(X_{1},X_{2}\right)$. The proofs of (83) and (84) are based on ([1], (31)) and ([1], (41)). The proof of (85) is similar to (82). We use one-time-pad coding for encoding $X_{1}$ instead of $X_{2}$. In this case. we send compressed $X_{2}$, ${\tilde{X}}_{1}$ and *U* over the channel, where ${\tilde{X}}_{1}$ is the output of the one-time-pad coding. We then have

$$\begin{array}{cc} \; & L^{b}(P_{XY},|X_{1}\left|,\epsilon \right)\leq \\ & \;\;\;\;\sum_{x\in X}\;H\left(Y|X=x\right)\;+H\left(X_{2}\right)\;+\;2+\;\left\lceil \log(\right|X_{1}\left|)\right\rceil . \end{array}$$

### Appendix B.5. Proof of Theorem 11

Let U=C(Y,W) be the output of the encoder. The code is bounded ϵ-private, thus $I(U;X_{1},X_{2})\leq \epsilon$. Due to recoverability, i.e., given the code, the shared secret key, and the private data, we can recover the useful data, which also satisfies $H(Y|W,U,X_{1},X_{2})=0$. Using (A10), we have

$$\begin{array}{cc} H(U) & \geq I(U;Y,W)\\ & =I(U;X)+H(Y|X)+H(W|X,Y)\\ & -H(W|X,U)-H(Y|X,U,W)-H(X|Y,W)\\ & +H(X|W,Y,U)\\ & \geq H(Y|X)+H(W)-H(W|X,U)\\ & -H(X|Y)+H(X|Y,U,W)\\ & \geq H(Y|X)-H(X|Y), \end{array}$$

where we used I(U;X)≥0, H(X|Y,U,W)≥0 and the independency of *W* and (X,Y). Proof of (87) is similar to (77).

### Appendix B.6. Proof of Theorem 13

To prove (91), let *U* be produced based on FRL ([1], Lemma 1) with assignments $X\leftarrow (X_{1},X_{2})$ and Y←Y, and encode it using any traditional code which uses at most H(U)+1 bits. Thus, $I(U;X_{1},X_{2})=0$ and $H(Y|X_{1},X_{2},U)=0$. Since $X_{2}=f\left(X_{1}\right)$, we have

(A15) $$\begin{array}{c} H\left(Y|X_{1},X_{2},U\right)=H\left(X_{1},U\right)=0. \end{array}$$

Using ([1], Lemma 2), we have

$$\begin{array}{c} H\left(U\right)\leq \sum_{x}H\left(Y|X=x\right). \end{array}$$

We encode $X_{1}$ by one-time-pad coding, which uses $\left\lceil \log(\right|X_{1}\left|)\right\rceil$ bits. Let the output of one-time-pad coding be ${\tilde{X}}_{1}$. We send compressed ${\tilde{X}}_{1}$ and *U* over the channel. Using the upper bound on H(U) and ${\tilde{X}}_{1}$, we have

$$\begin{array}{c} L^{b}(P_{XY},|X_{1}\left|,0\right)\leq \sum_{x\in X}\;\;H\left(Y|X=x\right)+1+\left\lceil \log(\right|X_{1}\left|)\right\rceil . \end{array}$$

On the receiver side, using the shared key, first $X_{1}$ is decoded and we then use (A15) to decode *Y* using $X_{1}$ and *U*. For the leakage constraint, the independency between ${\tilde{X}}_{1}$ and $X_{1}$ implies $I(X_{1},X_{2};{\tilde{X}}_{1})=0$, and the later follows since $X_{2}=f\left(X_{1}\right)$. Furthermore, we choose ${\tilde{X}}_{1}$ to be independent of *U*. Thus,

$$\begin{array}{c} I\left({\tilde{X}}_{1},U;X_{1},X_{2}\right)=I\left({\tilde{X}}_{1},U;X_{1}\right)=I\left({\tilde{X}}_{1};X_{1}\right)=0. \end{array}$$

The bounds (92) and (93) can be obtained using ([1], (31)) and ([1], (41)).
